# Selection of Specific Aptamer against Rivaroxaban and Utilization for Label-Free Electrochemical Aptasensing Using Gold Nanoparticles: First Announcement and Application for Clinical Sample Analysis

**DOI:** 10.3390/bios12100773

**Published:** 2022-09-20

**Authors:** Rokhsareh Ebrahimi, Abolfazl Barzegari, Reza Teimuri-Mofrad, Houman Kholafazad Kordasht, Mohammad Hasanzadeh, Maryam Khoubnasabjafari, Vahid Jouyban-Gharamaleki, Abbas Afrasiabi Rad, Nasrin Shadjou, Mohammad-Reza Rashidi, Mohammad Reza Afshar Mogaddam, Abolghasem Jouyban

**Affiliations:** 1 Student Research Committee, Tabriz University of Medical Sciences, Tabriz 5165665811, Iran; 2 Pharmaceutical Analysis Research Center, Tabriz University of Medical Sciences, Tabriz 5165665811, Iran; 3 Medicinal Chemistry Department, Faculty of Pharmacy, Tabriz University of Medical Sciences, Tabriz 5165665811, Iran; 4 Research Center for Pharmaceutical Nanotechnology, Biomedicine Institute, Tabriz University of Medical Sciences, Tabriz 5165665811, Iran; 5 Department of Organic and Biochemistry, Faculty of Chemistry, University of Tabriz, Tabriz 5166616471, Iran; 6 Biotechnology Research Center, Tabriz University of Medical Sciences, Tabriz 5165665811, Iran; 7 Nutrition Research Center, Tabriz University of Medical Sciences, Tabriz 5165665811, Iran; 8 Tuberculosis and Lung Disease Research Center, Tabriz University of Medical Sciences, Tabriz 5165665811, Iran; 9 Department of Anesthesiology and Intensive Care, Faculty of Medicine, Tabriz University of Medical Sciences, Tabriz 5165665811, Iran; 10 Kimia Idea Pardaz Azarbayjan (KIPA) Science Based Company, Tabriz University of Medical Sciences, Tabriz 5165665811, Iran; 11 Cardiovascular Research Center, Tabriz University of Medical Sciences, Tabriz 5165665811, Iran; 12 Department of Nanochemistry, Nanotechnology Research Center, Urmia University, Urmia 5756151818, Iran; 13 Food and Drug Safety Research Center, Tabriz University of Medical Sciences, Tabriz 5165665811, Iran; 14 Faculty of Pharmacy, Near East University, P.O. Box 99138 Nicosia, North Cyprus, Mersin 99138, Turkey

**Keywords:** rivaroxaban, aptamer, aptasensor, bioanalysis of drugs, biotechnology, advanced nanomaterial

## Abstract

For the first time, a novel aptamer was designed and utilized for the selective detection of rivaroxaban (RIV) using the integration of bioinformatics with biosensing technology. The selected aptamer with the sequence 5′-TAG GGA AGA GAA GGA CAT ATG ATG ACT CAC AAC TGG ACG AAC GTA CTT ATC CCC CCC AAT CAC TAG TGA ATT-3′ displayed a high binding affinity to RIV and had an efficient ability to discriminate RIV from similar molecular structures. A novel label-free electrochemical aptasensor was designed and fabricated through the conjugation of a thiolated aptamer with Au nanoparticles (Au-NPs). Then, the aptasensor was successfully applied for the quantitative determination of RIV in human plasma and exhaled breath condensate (EBC) samples with limits of detection (LODs) of 14.08 and 6.03 nM, respectively. These valuable results provide ample evidence of the green electrogeneration of AuNPs on the surface of electrodes and their interaction with loaded aptamers (based on Au-S binding) towards the sensitive and selective monitoring of RIV in human plasma and EBC samples. This bio-assay is an alternative approach for the clinical analysis of RIV and has improved specificity and affinity. As far as we know, this is the first time that an electrochemical aptasensor has been verified for the recognition of RIV and that allows for the easy, fast, and precise screening of RIV in biological samples.

## 1. Introduction

In clinical practice, atrial fibrillation (AF) is the most prevalent persistent heart arrhythmia disease. Stroke, thromboembolism, heart failure, and poor quality of life are all significant causes of death in people with AF and its possible complications [1,2,3]. There is an estimated increase in the risk of ischemic stroke of 15–20% with AF. The current therapeutic approaches to AF have substantial limitations, including low efficacy and a high risk of severe effects. Oral anticoagulants such as vitamin K antagonists (e.g., warfarin) reduce the incidence of stroke, but patients face a risk of substantial bleeding, and treatment success is hampered by dose changes and dietary compliance [4,5,6]. In recent years, several non-vitamin K direct oral anticoagulants (NOACs, e.g., dabigatran, edoxaban, apixaban, and rivaroxaban (RIV)) have been routinely administered to patients with AF. According to their safety profiles, all NOACs could have a lower risk of intracranial hemorrhage [7,8,9,10]. Furthermore, NOACs are appropriate warfarin alternatives for stroke prophylaxis in AF because they have fewer drug–food and drug–drug interactions [11]. According to previous reports, patients receiving RIV had statistically significant increases in major gastrointestinal bleeding when compared to patients taking NOACs or warfarin [12,13,14,15,16,17].

RIV, chemically identified as 5-chloro-N-({(5S)–2-oxo-3-[4-(3-oxomorpholin-4-yl) phenyl] 1, 3-oxazolidin-5-yl methyl) thiophene-2-carboxamide (Figure S1 (see Supporting Information)) is an oxazolidinone derivative anticoagulant that is increasingly being used to prevent blood coagulation following hip or complete knee replacement surgery [18,19,20].

In the pH range of 1 to 9, RIV is virtually insoluble in water and aqueous solutions and is prescribed in fixed doses (i.e., once daily) to all patients [21]. Rarely, 66% of RIV is cleared from circulation via glomerular filtration. Therefore, renal function plays an important role in its catabolism. Many clinical trials investigating RIV have used a creatinine clearance rate of less than 30 mL/min as an exclusion criterion. Accordingly, evaluation of the anticoagulant effect and concentration level of RIV in patients with renal impairment may be necessary to prevent drug overdose [22,23,24,25].

RIV is an oral, selective, and direct factor Xa (FXa) inhibitor, and it inhibits both free and bound FXa in the prothrombinase complex, with a rapid onset of action [26]. It has also been approved as a means of preventing and treating cardiovascular disease as well as thromboembolic disease, particularly stent thrombosis, angina pectoris, restenosis after angioplasty, or aortocoronary bypass; strokes; and atherothrombotic events in adult people with nonvalvular atrial fibrillation [18,27].

Although RIV has predictable pharmacokinetics and pharmacodynamics, there is still a strong tendency to measure and monitor RIV in some clinical circumstances (such as renal failure; during the control of therapy adherence; prior to surgery or planned invasive procedures in patients taking RIV; in the perioperative care of people who have received RIV; for patients who have had thromboembolic or bleeding episodes or if an overdose is suspected; in the elderly or in children; during bleeding or thrombotic episodes; and when treating physicians may want to know the RIV plasma level [28,29]). Therefore, to overcome the above challenges, the focus of scientists has been to design and develop a rapid and reliable diagnostic system to determine the level of RIV in clinical samples [30,31]. To ensure optimal performance while minimizing the risk of toxicity and other adverse effects, an accurate, sensitive, and efficient analytical procedure for the monitoring of RIV in clinical samples is highly demanded. For these conventional criteria, the laboratory tests that are employed (such as prothrombin time/international normalised ratio (PT/INR), or partial thromboplastin time-activated) are not suitable for anticoagulants with a specific aim [32,33,34]. The analytical approaches for evaluating RIV in biologic fluids that have previously been published are still restricted. These techniques include spectrophotometry [35], high-performance liquid chromatography (RP-HPLC) with UV [36,37,38], MS and MS/MS [30,38,39,40,41,42], and prothrombin time assay [43,44].

Although the above-mentioned methods provide simultaneous and accurate analytical results with low limits of detection (LODs) for RIV recognition, they do demand sophisticated instruments, tedious sample pre-treatment procedures, long processing times, high analysis costs foreach sample, complicated manipulation, and expert technical skills that restrict their application for the rapid monitoring of RIV [45,46]. As a result, there is a pressing need to establish a sensitive, specific, and field-portable method to detect RIV in clinical samples toward the early-stage diagnosis of heart disease and prevention in human bodies.

To achieve this goal, biosensors based on aptamers are presented as a promising platform that can identify different targets by producing a measurable physical signal. 

Aptamers are short and single-stranded DNA or RNA oligonucleotides that can selectively bind to a specific target, including proteins, peptides, carbohydrates, small molecules, toxins, and even live cells.

They can be used to detect disease-related molecular markers or can be used as medications, controlled drug release systems, and drug delivery mechanisms. [47,48]. They are efficient sensing instruments for detection, binding to their target with high specificity and affinity because of their three-dimensional shape [49,50]. Such oligonucleotides can be quickly synthesized through an in vitro selection procedure known as the systematic evolution of ligands by exponential enrichment (SELEX) [51,52]. They can also be easily modified and linked to certain functional groups. Because of their numerous distinguishing characteristics, such as stability, easy synthesis, controllable size, immunologically inert, and low dissociation constants (K_d_) (from picomolar to nanomolar), aptamers have become ultra-selective recognition elements compared to other existing molecules for biochemical assay. Aptamers have been widely used for various sensor-based approaches, such as in electrochemical, colorimetry, fluorescent surface, and plasma resonance-based biosensors [53]. Among these sensing technologies, electrochemical aptasensor-based methods have attracted more attention because of their advantages, such as simple instrumentation, rapidness, excellent compatibility with miniaturized technologies, and their remarkable sensitivity [54,55]. Keeping this in mind, in this report, we successfully synthesized the first aptamer and developed an innovative biosensor for the detection of RIV. RIV-specific aptamers were created via the SELEX method to screen a library of more than 10^14^ single-stranded DNA. In addition, a label-free electrochemical aptasensor was created as a biosensing platform for detecting RIV using this aptamer, which is capable of sensing RIV at the 1 nM level [56]. The aptamer was immobilized by a self-assembly procedure on the surface of ITO-PET modified by gold nanoparticles.

Among these, Au-NPs were chosen due to their distinct preparation characteristics, such as size distribution control, high stability, and biological compatibility, and ITO electrodes were selected because of their weak electrocatalytic activity and low background flow, which reduce noise, representing the best combination for electrochemical aptasensors [57,58,59]. 

Keeping this in mind, in this study, in order to overcome RIV’s absence of a sensing probe, we successfully designed and synthesized the first aptamer for the specific detection of RIV. RIV-specific aptamers (5′-TAG GGA AGA GAA GGA CAT ATG ATG ACT CAC AAC TGG ACG AAC GTA CTT ATC CCC CCC AAT CAC TAG TGA ATT-3′) were created via the SELEX method to screen a library of more than 10^14^ single-stranded DNA. To select aptamers against RIV, an improved SELEX technique was developed via in vitro selection utilizing affinity chromatography by immobilizing the ssDNA library on epoxy-activated Sepharose 6B beads.

Taken together, the proposed aptamer and the supporting sensing platform provide a useful tool for therapeutic RIV monitoring in biofluids from patients with arterial fibrillation. So, a new low-cost strategy with high repeatability and suitable sensitivity was prepared to achieve the label-free electrochemical aptasensing of RIV in real human plasma and exhaled breath condensate (EBC) samples.

For the first time, ITO-PET was modified by AuNP using a green electrogeneration strategy. Based on Au-S binding, the thiolated aptamer was immobilized on the surface of ITO-PET-modified AuNPs. So, the engineered interface was used for the sensitive and targeted monitoring of RIV in human real samples. 

Then, an ecofriendly approach was used for the recognition of RIV in the EBC and plasma samples of patients suffering from atrial fibrillation. It is expected that using this approach, we will be able to conduct real-time and non-invasive measurements of RIV in human biofluids.

## 2. Experimental Section

### 2.1. Reagents and Materials

RIV was purchased from the Abidi pharmaceutical company (Tehran, Iran). The ssDNA library (5′-TAG GGA AGA GAA GGA CAT ATG AT (N20 or N30 or N40) TTG ACT AGT ACA TGA CCA CTT GA-3′), forward primer (5′-TAG GGA AGA GAA GGA CAT ATG AT-3′), and reverse primer (5′-TCA TCA AGT GGT CAT GTA CTA GTC AA-3′) were obtained from Microsynth (Balgach, Switzerland). 

Silica gel, hexane 1, 6-diamine, sodium hydroxide, ethanol, ethyl acetate, hexane, methanol, acetonitrile, acetone, acetic acid, formic acid, hydrogen peroxide, dimethylsulphoxide (DMSO), dimethylformamide (DMF), and all of the other common solvents were purchased from Sigma Aldrich.

An indium tin oxide–poly polyethylene terephthalate (ITO-PET) electrode (0.5 mm × 2 mm, thickness of 0.7 mm, 15–20/cm^2^) was purchased from Woo-Yang Corp (Wuhan, China). NaCl, KCl, MgCl_2_, potassium ferrocyanide (K_4_[Fe(CN)_6_]·3H_2_O), potassium ferricyanide (K_3_[Fe(CN)_6_]).3H_2_O, anhydrous acetic acid, EDC/NHS (1-ethyl-3-(3-dimethylaminopropyl) the carbodiimide/N-hydroxysuccinimide), Tris-HCl (tris(hydroxymethyl)aminomethane hydrochloride), cetyltrimethylammonium bromide (CTAB), MCH (mercaptohexanol), H_2_SO_4_ (sulfuric acid), ascorbic acid, arginine, glucose, L-cysteine, glycine, tyrosine, uric acid, glutamic acid, magnesium, L-proline, hydrochlorothiazide, metoprolol, nitroglycerin, aspirin, and losartan and KCl (potassium chloride) were obtained from Merck (Darmstadt, Germany). 

Epoxy-activated Sepharose 6B and the columns were obtained from Thermo Scientific and were purchased from GE Healthcare (Piscataway, NJ, USA). The QIAEX II Gel extraction kit and QIAprep spin miniprep kit were purchased from QIAGEN Inc. (Valencia, CA, USA). Low-melting-point agarose was obtained from Fermentas (Burlington, ON, Canada), and the pGEM^®^-T vector was purchased from Promega (Madison, WI, USA). PCR master mix (2×) and the DNA ladder were purchased from the company Amplicon. Deionized and pyrogen-free water was obtained from a Milli-Q water purification system (Millipore, Washington, DC, USA). Nuclease-free water was used to make all of the aqueous solutions. All chemicals were at the maximum purity obtainable unless otherwise noted.

Deionized water and human plasma samples were prepared by the Ghazi Pharmaceutical Company (Tabriz, Iran) and the Blood Transfusion Research Center (Tabriz, Iran), respectively. Sample donors signed a written consent form approved by the ethics committee of the Tabriz University of Medical Science (IR.TBZMED.VCR.REC.1397.317).

### 2.2. Instrumentation

(ATR-FTIR) Analysis: Attenuated total reflection Fourier transform infrared (Bruker Vertex 70 ATR Model) was used to monitor the functional groups on modified ITO-PET electrode surfaces using an ATR apparatus. The measurements were recorded in the range of 400–4000 cm^−1^ in order to investigate the nature of the chemical bonds. 

The morphology of the bulk materials and modified ITO-PET electrode surfaces were characterized by a high-resolution field emission scanning electron microscope (FE-SEM, HitachiSU8020, Praha, Czech) with an operating voltage of 3 kV, and the chemical compositions of the electrodes were analyzed by energy dispersive spectroscopy (EDX) coupled with FE-SEM equipment.

AFM Analysis: Atomic force microscopic analyses were recorded using Nanosurf (AG Gräubernstrasse 124410 Liestal, Switzerland) in tapping mode to confirm the surface immobilization of the aptamers on the substrates. The scan speed was 2 μm.s^−1^, with a resolution of 256 pixels per line. Electrochemical measurements were carried out in an ordinary three-electrode cell (from Metrohm, The Netherland) powered by an electrochemical system comprising a Palm Sense 4c system with PS4.F1.05 (Palm instruments, Utrecht, The Netherlands). The three-electrode system consisted of Ag/AgCl-saturated KCl (from Metrohm, Netherland) as a reference electrode, a platinum wire, and a modified ITO-PET electrode with Au nanoparticles (2 mm × 20 mm), which were utilized as the counter and working electrodes, respectively. Chronoamperometry (ChA) and square-wave voltammetry (SWV) analyses were performed in the presence of 0.01 mM of K_3_ [Fe(CN)_6_]/K_4_ [Fe(CN)_6_] (1:1) mixture and 0.1 M KCl. For the ChA measurements, t_equilibrium time_ = 2, interval time = 0.1 s, run time = 200 s, and E_DC_ =0 V were employed. In SWV technique investigations, the scan rate was adjusted for 0.05 V s^−1^, the modulation amplitude was 0.25 mV, the step potential was 0.01 mV, and the potential range was −1 V to 1 V. All measurements were the average of at least three replicates.

The quantity and quality of the isolated DNA were analyzed using a Nanodrop 1000 (NanoDropND-1000 spectrophotometer; Thermo Fisher Scientific, Waltham, MA, USA). Chromatographic analysis was performed using Alliance separations module 2795 (Waters, Milford, MA, USA), which consisted of a quaternary solvent delivery system with a vacuum degasser pump with a C18 column (60 × 4.6 mm × 5 microns) from the Agilent Company. The confirmatory analysis of the blank sample was performed on the same apparatus coupled with a Quattro Micro quadrupole mass spectrometer (Waters-Micro mass, UK) equipped with an electrospray source (Z-spray). During the analysis of the samples, the flow rate of the mobile phase (methanol: water at a ratio of 65:35) was adjusted to 0.6 mL/min. SPR measurements were conducted using a SRPNavi 210A device (BioNavis Ltd., Tampere, Finland) with gold chips (BioNavis Ltd., Helsinki, Finland).

### 2.3. Preparation of RIV Affinity Matrix 

#### 2.3.1. Synthesis of Acidic Hydrolysis Product of RIV 

Synthesis of the acidic derivative of RIV was performed based on the previously reported method [60]. For this, RIV (0.1 g, 0.045 mmol) was added to a round-bottom two-necked flask with a mechanical stirrer and reflux condenser. Acetic acid (0.2 g), concentrated hydrochloric acid (0.6 g, 37%), and 0.1 g of water were added to the flask. The suspension was stirred for 5 to 6 h at 70 ° C. 

After the completion of the reaction, the suspension was kept at room temperature for 15 h. The resulting crystals were filtered and washed by suction with 0.08 mL of acetic acid. For better filtration, crystals were suspended twice in 0.3 mL of isopropanol and were washed again with a suction filter and again with 0.4 mL of isopropanol. The remaining crystals remained at 35 °C for 15 h at a pressure of less than 80 mbar to obtain a pure product as a light brown solid. Yield: 0.93 g (85%). ^1^H NMR (400 MHz, DMSO): δ (ppm): δ = 3.43 (m, 2H), 3.64 (m, 2H), 3.76 (m, 2H), 3.90 (m, 1H), 4.2 (m, 2H), 4.12 (s, 2H), 4.20 (m, 1H), 4.86 (m, 1H), 7.21 (d, 1H), 7.38 (br.m, 2H), 7.58 (m, 2H), 7.77 (d, 1H), and 9.12 (m, 1H).

#### 2.3.2. Substitution Epoxy-Activated Sepharose 6B with Hexandiamine

The amination of epoxy-activated Sepharose 6B was performed in the presence of hexamethylene diamine according to the method of Teng et al. [61]. Under vacuum, a fritted glass funnel was used to filter the epoxy-activated agarose and was rinsed with 200 mL deionized water. Hexane-1, 6-diamine (excess of 10 M to free epoxy groups on the gel) dissolved in water was added to the epoxy-activated Sepharose 6B, and the suspension was then slowly spun at 25 ° C for 18 h. Under these conditions, the amino group of the hexanediamine binds covalently to the epoxy adduct on Sepharose 6B. The 6-Aminohexyl-activated Sepharose 6B was washed generously with water and was transferred to bottles. 

#### 2.3.3. Immobilization of RIV Acidic Derivative on the Substituted Sepharose 6B

First, deionized water was used to wash the replaced Sepharose. A mixture of RIV acidic derivative (8.6 mmol) and carbonyldi-imidazole CDI (0.8 mmol) in 2 mL of DMF were stirred for 1 h at room temperature with a magnetic stirrer under argon gas [62]. Then, the magnetic stirrer was removed from the reaction medium. After that, the dry substituted Sepharose (1 g) was added to the reaction media and shaken overnight. Under these conditions, the carboxyl groups of the RIV acidic derivative link covalently to the free terminal amino groups of the hexanediamine. At the end of the reaction, the Sepharose 6B-(RIV acidic derivative) gel was washed with a large amount of deionized water (3 × 100 mL) in a sintered glass funnel and was then centrifuged. Finally, the solvent was vacuumed out of the mixture after it was filtered.

#### 2.3.4. Characterization of RIV–Sepharose Support

The Fourier transform infrared spectra (FT-IR) of the RIV derivatized gel and of the epoxy-activated Sepharose (with no RIV) were acquired on a Thermo Scientific Nicolet iS10 FT-IR spectrometer (Thermo Scientific, Waltham, MA, USA). Spectra were recorded at 4 cm^−1^ (128 scans) over the 400–4000 cm^−1^ range, with and without baseline corrections. Scanning electron microscopy (SEM) images of both the derivatized RIV gel and epoxy-activated Sepharose were acquired in a Hitachi S-2700 with a UHV Dewar detector (Rontec EDX) (Tokyo, Japan). The samples were magnified 150, 450, and 4000×. Before all of the analyses, the samples were well-dried at 50 °C in the presence of phosphorus pentoxide.

### 2.4. Generation of Random Library and Primers

The random DNA library contained central randomized sequences of 20, 30, or 40 nucleotides (N20 or N30 or N40) flanked by a 23-nucleotide constant region: 5′ -TAG GGA AGA GAA GGA CAT ATG AT(N20 or N30 or N40) TTG ACT AGT ACA TGA CCA CTT GA-3′. Strand primer up (5′-TAG GGA AGA GAA GGA CAT ATG AT-3′) and a3′ (-) strand primer down (5′-TCA AGT GGT CAT GTA CTA GTC AA-3′) were prepared using Micro synth AG (Balgach, Switzerland). The library was used as a template DNA for the synthesis of single-stranded DNA (ssDNA) in asymmetric PCR.

### 2.5. Selection of Specific for Aptamer Sequences RIV Based on ssDNA Library Immobilized SELEX

The special aptamers for RIV were selected and extracted at the end of the SELEX cycles by affinity chromatography on a RIV–Sepharose column (Scheme S1 (see Supporting Information)). Before each selection round, the RIV-modified affinity column was washed (3 times) with the binding buffer (20 mM Tris-HCl, 50 mM NaCl, and 5 mM MgCl_2_, pH = 7.4). Next, 50 μL (50 pmol/μL) of the synthetic ssDNA library was dissolved in 200 μL of binding buffer and was slowly denatured at 95 °C for 10 min before being quickly cooled on ice for 10 min. Then, the appropriate quantity of RIV-immobilized beads (2 mL) was added to the above hybrid library solution and incubated with gentle agitation (1 h at room temperature) for immobilization. The medium was washed with wash buffer (20 mM Tris-HCl, 50 mM NaCl, and 5 mM MgCl_2_, 0.01% Tween 20, pH = 8.0) to remove the unspecific and unbound DNA. Additionally, the bound ssDNAs were then eluted in binding buffer with four column volumes with 5 to 0.5 mM of RIV. The recovered ssDNA was amplified by asymmetric PCR. The 25 μL PCR amplification was made of 10 pg ssDNA template, forward primer (0.4 μM), reverse primer (0.004 μM), and 10 µL PCR master mix (2×). 

The PCR cycling conditions were 94 °C for 5 min followed by 40 cycles of 94 °C for 30 s, 56 °C for 30 s, 72 °C for 30 s, and a final step of 72 °C for 5 min. To verify the amplification and ssDNA extraction, PCR production was electrophoresed in 3% agarose gel. After staining the gel in safe stain SYBR green, the ssDNA bands were cut under UV light and were purified using a gel extraction qiagene kit. The purified DNA was finally quantified using a ND-2000 spectrophotometer. The amount of separated ssDNA was used for the next nine rounds to further enrich the aptamers, and in the end, ssDNA was used as a template in routine PCR with equal concentrations of primer for the amplification of dsDNA. The amplified dsDNA was cloned into the PGEM vector according to the manufactured product and was transformed into *E. coli* bacteria. Plasmid extraction was conducted according to the qiagene miniprep kit protocol. After controlling for positive clones by PCR, the samples were sent to be sequenced by macrogene Korea [63].

### 2.6. Sequence Analysis 

The obtained sequences were aligned using clustal X software to perform homologous comparisons and to specify the selected DNA aptamers. 

### 2.7. Affinity Assay and Kd Measurements Using Surface Plasmon Resonance (SPR)

The four selected aptamers were synthesized by Bioneer (Korea), and the affinity for their target RIV was determined using an SPR assay. Briefly, after activation of the Au chips with 0.05 M of EDC/NHS solution for 7 min, they were washed 3 times with PBS and coated with 25 µg/mL of aptamers in acetate buffer (pH = 5) for one hour. All measurements were performed at a fixed angle wavelength at room temperature (25 °C), and during all of the SPR experiments, the flow of the solution was kept constant at a rate of 20 μL min^−1^. The dissociation constants (KD) of the thiolated aptamers were obtained in combination with a concentration series of the target RIV (20–500 nM), and the saturation curves were obtained by plotting the amount of target-bound aptamers against the corresponding RIV concentration. Data were analyzed using data viewer and TraceDrawer™ 210A SPR Bionavis software (Helsinki, Finland).

### 2.8. Preparation of Plasma Samples

The Iranian Blood Transfusion Research Center (Tabriz, Iran) provided human plasma and blood samples. Aliquots of plasma were put in microtubes and were kept at −20 °C until analysis. Whole blood samples were also preserved at 4 °C in citrated tubes. Frozen human plasma samples were thawed at room temperature and were vortexed on a daily basis to ensure uniformity. After gently thawing the samples, RIV was added to 0.5 mL of an aliquot volume of this sample. To extract the plasma, 0.5 mL of plasma was added to 0.5 mL of acetonitrile and was vortexed for 2 min. It was then centrifuged for 3 min at 12,000 rpm. Then, roughly 0.5 mL of the extracted plasma supernatant was removed and added to the support electrolytes. 

### 2.9. Preparation of EBC Samples

EBC samples were acquired from healthy volunteers utilizing a lab-made apparatus based on a patented Iranian cooling trap technique. The manufactured device provides a wide range of temperatures from 0 to 25 °C and can rapidly condensate exhaled air immediately after breathing. EBC samples collected from a healthy volunteer were transferred to a 0.1 mL volumetric flask, a standard solution with the desired concentration was added up to the marked line, and electrochemical analysis was performed.

### 2.10. Chromatographic Conditions

Chromatographic separation was performed on an Agilent C18 column (60 × 4.6 mm, 5 μm particle size) at a column temperature of 35 °C. Mobile phase A was HPLC grade water, and mobile phase B was methanol. Phases were added at a ratio of 65:35. The flow rate of the mobile phase was 0.6 mL/min. The injection volume was 20 μL, and the detection was carried out at 254 nm. Water and acetonitrile 10:90 (*v*/*v*) were used as a diluent. Stock solutions of RIV (2.0 mg/mL) were prepared by dissolving 200 mg of RIV standard in a minimum amount of DMSO and were made up to volume with diluents to obtain a concentration of 2.0 mg/mL. The analysis was performed in positive electrospray ionization mode ESI+: capillary voltage, 3.5 kV; cone, 35 V; extractor, 1 V; RF lens, 0 V; source temperature, 100 °C; desolvation temperature, 300 °C; desolvation gas flow rate, 600 L/h; and curtain gas (nitrogen 99.99% purity) flow, 50 L/h.

## 3. Results and Discussion

### 3.1. RIV–Sepharose Support Synthesis 

The main aim of the present study was to develop an electrochemical-based aptasensor method for the determination of RIV in plasma and EBC. To achieve this purpose, we synthesised the first single-standard DNA aptamer for binding to RIV using the SELEX method. RIV was linked to epoxy-activated Sepharose 6B via a hexandiamine intermediate linker, which was made possible by constructing a novel ligand matrix for affinity chromatography. The schematic representation of the mechanism of the immobilization process of RIV on the substituted Sepharose 6B is shown in Figure S2.

In the first step, in order to couple RIV to Sepharose 6B, the oxomorpholin ring of RIV was opened, and a carboxylic acid derivative was synthesized. Significant degradation of the oxomorpholin ring of RIV and the synthesis of its carboxylic acid derivative (compound **1**) were reported in acidic and basic hydrolytic stress conditions [64]. Due to the ease and convenience of the acidic hydrolytic method, we used it in this work. For the synthesis of compound **1**, RIV was degraded in a solution composed of acetic acid, hydrochloric acid, and water at 70 °C. The degradation product was characterized using NMR and FT-IR.

In the second step, to facilitate the covalent link between carboxylic (compound **1**) and epoxy (Sepharose), we used a hexamethylene diamine linker, which contained amine groups. The epoxy-activated Sepharose 6B was reacted with hexanediamine at room temperature for 18 h on a rotary shaker, as described previously by Teng et al. [61].

Finally, the substituted Sepharose 6B compound (**2**) was conjugated with a carboxylic group (COOH) of compound (**1**) through its hexamethylenediamine spacer (NH_2_ (CH2)_6_NH_2_).

CDI (1, 1′-carbonyldi-imidazool) was used to mediate the coupling reaction for carboxylic activation; afterwards, the reaction between compound (**1**) and compound (**2**) resulted in the synthesis of the final RIV–Sepharose at RT temperature by mild shaking (Figure S2). The structure of RIV–Sepharose was studied and was completely characterized using Fourier transform infrared spectroscopy (FTIR) and field emission scanning electron microscopy (FESEM).

### 3.2. Characterization of RIV–Sepharose

Figure S3 depicts the FT-IR spectra of the RIV acidic derivative. The corresponding FT-IR spectra of the RIV and compound (**1**) showed an additional stretching peak at 1717 cm^−1^ and 3200–3700 cm^−1^, respectively, corresponding to C=O (COOH) and OH (compound **1**). The two absorption peaks observed at 1717 cm^−1^ and 1745 cm^−1^ (Figure S3B) are related to the C=O bonds carbamate and carboxylic acid, respectively. The molecular structure of RIV’s acidic derivative can be well assigned in the proton 1H NMR spectra (Figure S4 (see Supporting Information)). When comparing the NMR spectra of the RIV acidic derivative with those in the references, it was confirmed that this molecule had been synthesized [60,65].

The coupling results were confirmed using the ninhydrin assay and FT-IR. The Sepharose hexanediamine-coupled beads to ninhydrin solution, and after 2 min, the color of the solution turned purple. In theory, ninhydrin reacts with Ruhemann’s purple complex when bound to free amino residues (primary amines) [66]. Indeed, ninhydrin has the ability to attach to primary amine groups on epoxy-activated Sepharose 6B beads. The existence of the absorption peak at 3444 cm^−1^ confirms the presence of amine groups in the FT-IR spectra of compound (**2**).

The FT-IR spectra of the derived Sepharose were compared with the epoxy–Sepharose spectra, and hexanediamine–Sepharose was used to test the affinity ligands’ immobilization on the matrix (a). Support for hexanediamine–Sepharose (b) and RIV–Sepharose support (c) is presented in Figure S5.

When compared to the FT-IR spectrum of blank Sepharose, the RIV–Sepharose spectra indicated that there were two additional apparent adsorption bands at 1651 and 1732 cm^−1^ related to the C=O stretching vibration in RIV. Because this band does not exist in epoxy–Sepharose or in the hexanediamine–Sepharose spectrum, it is a qualitative indication of successful coupling between the affinity of the RIV ligand and the Sepharose matrix successfully establishing a covalent bond between Sepharose and RIV.

FE-SEM (micrographs with 2, 5, 10, and 50 magnification) was employed to investigate the surface morphology and structure of the Sepharose beads before and after the immobilization process, and no significant alterations were detected between them (Figure S6). This finding reveals that Sepharose retains its original morphology and physical qualities after being subjected to the curing process, which is critical for its use as a chromatographic substrate [67,68,69].

### 3.3. Selection of Specific Aptamers for Detection of RIV

The RIV-bound aptamers with high affinity and selectivity were enriched through the repeated selection method. A total of nine consecutive in vitro selection cycles were carried out to extract the ssDNAs that bind to RIV-modified epoxy-activated Sepharose 6B. Due to the saturation of the binding sites on the matrix, the amount of RIV bound ssDNA retrieved after the ninth cycle remained the same as in the tenth cycle, and the PCR band of cycle nine was brighter than that of the other selection cycles.

**Scheme 1:**
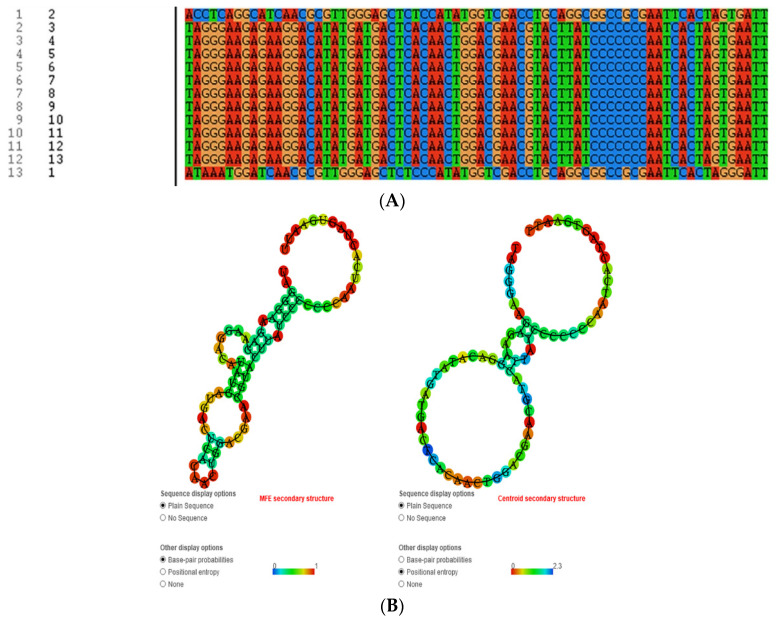
(**A**) The alignment of RIV-specific DNA aptamers. (**B**) The 76-mer ssDNA aptamer’s secondary structure. M-Fold software used the free minimization approach to anticipate the structure. The likelihood is shown by the color annotation.

Finally, the ten-cycle asymmetric PCR products were cloned into the Pgem vector. It is important to point out that the 13 clones that were positive at the blue/white screening were picked up and sequenced. Scheme 1A shows the alignment of the selected aptamers.

The majority of the selected clones showed a common sequence: 5′-TAG GGA AGA GAA GGA CAT ATG ATG ACT CAC AAC TGG ACG AAC GTA CTT ATC CCC CCC AAT CAC TAG TGA ATT-3′. This sequence, named “RIV aptamer,” was chosen as the aptamer RIV. The secondary structure of the selected sequence was predicted using M-Fold software, as shown in Scheme 1B.

### 3.4. Affinity Constants

The affinity constants of the RIV aptamers were determined using SPR in the range of 1.56 × 10^−9^ to 8.84 × 10^−9^ nM, and the aptamer used in this study with the lowest affinity constant was determined to be the premier aptamer due to its better binding affinity towards RIV (Figure S7).

### 3.5. Fabrication of RIV Aptasensor

**Scheme 2:**
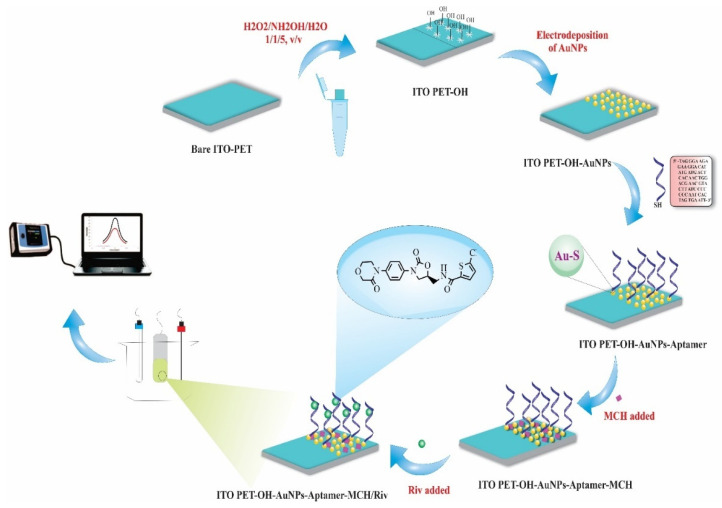
The preparation and operation of the developed aptasensor for detection of RIV.

Scheme 2 depicts the manufacturing processes of the electrochemical aptasensor platform for RIV detection. All of the electrochemical studies were carried out using an ITO-PET electrode as the working electrode, a Pt wire as the counter electrode, and Ag/AgCl as the reference electrode. The ITO-PET/ITO-PET-OH/ITO-PET-OH-AuNPs/ITO-PET-OH-AuNPs-aptamer and ITO-PET-OH-AuNPs-aptamer/RIV electrodes were utilized as working electrodes during electroanalysis. After each incubation period, the electrode surface was rinsed with deionized water.

To obtain the RIV aptasensor, the ITO-PET electrode surface was modified in multiple stages. Firstly, ITO-coated PET film was cut into individual pieces (20 mm × 5 mm), followed by the removal of the protective film. Then, they were sonicated in acetone for one minute in order to clean the surface of the ITO-PET sheets and were subsequently dried after rinsing with ultrapure water by streaming N_2_ immediately. After this process, for hydroxyl ends to form on the ITO-PET electrodes and to remove particulate contaminants and gain a hydroxylated active ITO-PET electrode surface, the substrates were placed in NH_4_OH: H_2_O_2_: H_2_O (1:1:5, *v/v/v*) solution for 90 min at room temperature to remove contaminants. After this process, the electrodes were then rinsed in ultrapure water and dried in a N_2_ stream after this process. Afterward, to obtain an identical working area, a piece of insulating adhesive tape was tightly wrapped around the ITO-PET electrode [70].

Following the formation of OH groups on the surface of ITO-PET, green electrogeneration was subsequently performed on the AuNPs on the surface of ITO-PET-OH. The correct binding of sensing biomolecules (such as aptamers, DNA, enzymes, and antibodies) to the electrode without affecting their immunological activity and specificity is another critical issue for selective detection. For the detection of the target biomarkers, loose binding may result in erroneous positive or negative results [71]. Due to the exceptional physical and chemical properties arising from their diverse size, shape, morphology, crystal orientation, porosity nature, and high and advantageous surface areas, nanostructures such as noble metal nanorods, nanowires, nanoflowers, etc., could be used for particular electrical, optical, thermal, catalytic, or magnetic functions [72]. Among the noble metals, AuNPs are also of high importance due to their unique properties during bio-assay, such as size, collective effects, morphology, electronic properties, high surface-to-volume ratio, great biocompatibility, low toxicity, and local dielectric properties, which make them a significant tool in nano-biotechnology [73].

Interestingly, the green electrogeneration of AuNPs on various surfaces is an intriguing technology that has the advantages of high controllability, being a single-step process, and being simple to control [74,75]. Based on these interesting properties, the electrodeposition of AuNPs in the presence of CTAB/Arg as a template to control its morphology is important for the suitable immobilization of aptamers and to enhance aptasensor performance for the efficient detection of RIV in complex matrices. In this investigation, coating was carried out in one step, and CTAB and arginine–AuNPs were utilized to boost the sensitivity and specificity of the biosensors. CTAB can be deposited on electrode surfaces utilizing micellar electrodeposition as an adsorbent. As a result, a proposed mechanism for the production of gold nanostructures incorporates the following stages: (a) a micellar Au-CTAB complex is adsorbed on the electrode surface; (b) during cathodic scan, “hemi-micelles” containing gold nanostructure form on the electrode surface as a result of electrochemical reduction [73,76,77,78,79].

So, the ChA technique was employed for the electrodeposition of the AuNPs on the surface of the Au electrode in the solution containing 0.01 M HAuCl_4_·3H_2_O and 0.01 M CTAB and arginine (0.003 g). The sweep rate was 200 mV/s, and the number of cycles was 10. As displayed in Figure S8, using this technique, the Au nanolayer was formed using a one-step method (200 s and E = 0.0 V). After the preparation of this interface (ITO-PET/OH/AuNPs electrode), the isolated aptamer was immobilized on the surface of the ITO-PET/OH/Au NPs electrode via a self-assemble strategy, and for this purpose, 5 μL of DNA- 5′ SH TAG GGA AGA GAA GGA CAT ATG ATG ACT CAC AAC TGG ACG AAC GTA CTT ATC CCC CCC AAT CAC TAG TGA ATT-3′ was immobilized on the surface of the electrode and was incubated for 20 min at room temperature. Then, MCH was added as a blocking agent for the sites of the electrode that had not reacted with aptamer for 20 min to reduce the nonspecific adsorption of the analyte and to ensure that the aptamer was only attached to the surface of the AuNPs-modified ITO-PET via a sulfide bond.

During the last step of fabrication, after being properly washed with ultrapure water, the acquired ITO-PET-OH-AuNPs-aptamer-MCH-functionalized ITO substrate was ready for RIV measurement. Finally, the prepared bioelectrode was used for RIV detection. Electrode preparation steps, optimization studies, and the analytical behavior of developed aptasensor were studied by following the electrochemical CV technique. All of the electrochemical experiments were performed in a solution containing K_3_[Fe(CN)_6_]/K_4_[Fe(CN)_6_] and KCl (0.01 M) (1:1) as a redox probe. In the CV experiments, the working potential range was between −500 and 500 mV at a scan rate of 100 mVs^−1^.

### 3.6. Electrochemical Behavior of the Engineered Aptasensor

**Figure 1:**
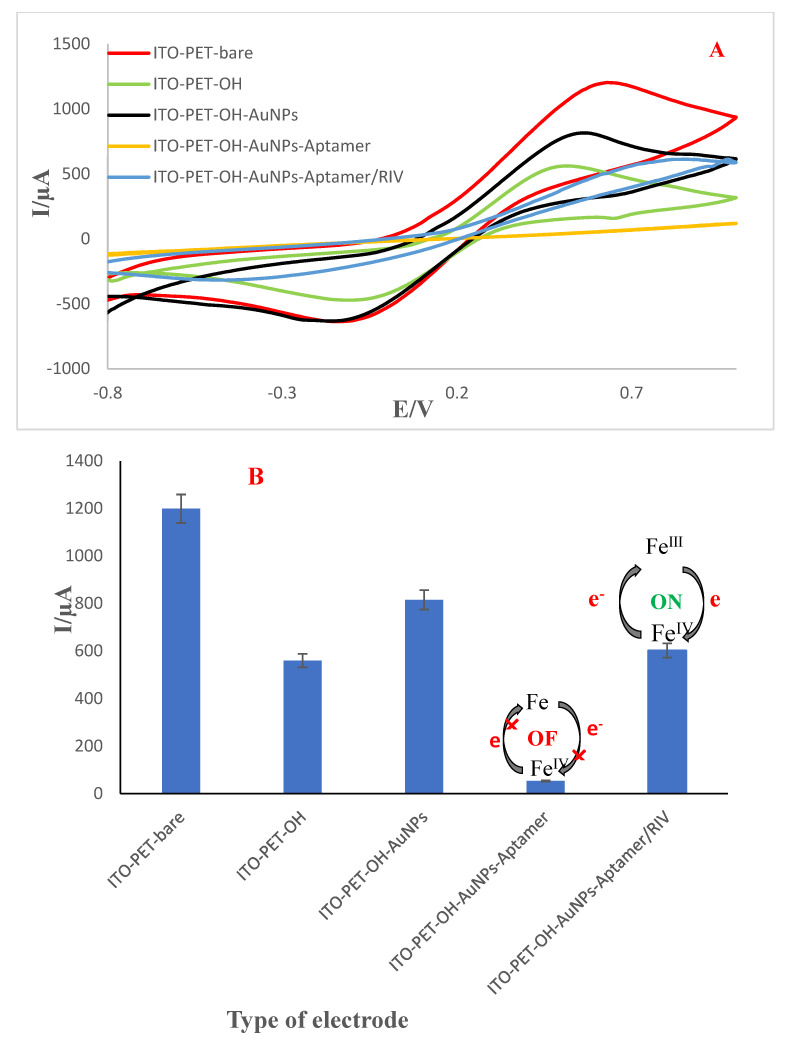
(**A**) CVs of bare ITO-PET, ITO-PET-OH, ITO PET-OH-AuNPs, ITO PET-OH-AuNPs-aptamer, and ITO PET-OH-AuNPs-aptamer/RIV in the presence of ferrocyanide/ferricyanide/KCl (0.01 M) at the potential range of −1 to +1 V and at a sweep rate of 100 mVs^−1^. (**B**) Histogram of peak currents versus the type of electrode. (n = 3).

The electrochemical behavior of the aptasensor preparation steps was clearly investigated via the CV technique in 0.01 M of ferrocyanide/ferricyanide containing KCl solution as a mediator, as it provides a simple and effective method for evaluating electron transport between the solution and the electrode surface. As shown in Figure 1A, K_3_[Fe(CN)_6_] and K_4_[Fe(CN)_6_] showed almost completely irreversible behavior on the (a) bare ITO-PET, (b) ITO-PET-OH, (c) ITO-PET-OH-AuNPs, (d) ITO-PET-OH-Au NPs-aptamer, and (e) ITO-PET-OH-Au NPs-aptamer /RIV (1 mM) electrodes when a potential sweep rate of 100 mVs^−1^ had been employed. According to the obtained results, the oxidation peak of the EC probe on the surface of ITO-PET has an intensity of 1198.74 µA, showing a higher oxidation peak current than the other electrodes. When the bare electrode was modified by OH, the peak current dropped greatly (559.3 µA) and validated the surface-blocking redox process’ hindrance behavior [Fe ^(III)^/Fe ^(IV)^]. After the gold nanoparticles were electrogenerated on the surface of ITO-PET-OH, the peak potential shifted to 0.549 V, and the peak current increased to 814.89 µA. This result shows that the electrodeposition of the Au NPs on the surface of ITO PET-OH and the excellent features of Au NPs, such as electrical conductivity and a large specific surface area, allowed more (Fe(CN)_6_)^3−/4−^ to reach its surface and electron transfer to occur at a higher rate. Certainly, the Au nanoparticles stabilized on the ITO-PET-OH film reveal appropriate catalytic characteristics as well as increased the rate of electron transfer and demonstrated appropriate electroactivity. Therefore, the gold nanoparticles are promising candidates for forming an aptamer-active substrate.

In contrast, when the aptamers were assembled on the ITO PET-OH-modified electrode via the AuNPs reaction, the peak currents of ITO PET-OH-AuNPs-aptamer obviously decreased, which led to a decrease in the peak height (from 0.5 to 0.6 V and from 814.89 to 53.16 μA in DPV the plots). This result indicates that as a biological element, aptamers (bioreceptors) were successfully immobilized on ITO-PET-OH-AuNPs. Because aptamers are macromolecule and occupy a large portion of the electrode surface, it reduces the electron transfer of ferro/ferry (supporting electrolytes). In reality, aptamers function as an insulator, slowing down electron transmission.

According to the findings, in the presence of RIV targets, it was observed that the anodic peak current increased to about 601.36 µA. This indicates that in the presence of RIV, as a result of the formation of a RIV/aptamer complex on the surface of the electrode, the aptamer folds due to RIV binding; therefore, the presence of a higher negative charge (both of the aptamer phosphate group and the AuNPs) resulted in a larger repulsion of [Fe(CN)_6_]^3−/4−^ at the electrode surface, and the interfacial charge transfer was increased. This means that the aptamer has completely covered the electrode’s surface and that the signal is set as Signal ON.

As a result, it is clear that the developed aptasensor is capable of successfully detecting RIV. Figure 1B shows a histogram of the peak currents during different modification steps. The prepared biosensor was characterized using FE-SEM, Atomic Force Microscopy (AFM), and ATR-FTIR (Figures S10–S12).

### 3.7. Analytical Performance of Aptasensor towards Detection of RIV

**Figure 2:**
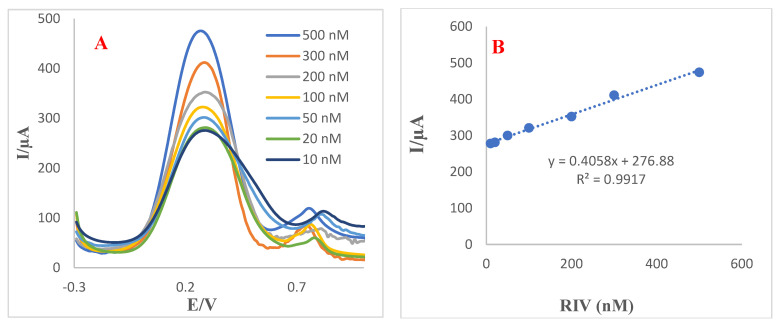
(**A**) SWVs of the engineered aptasensor ITO-PET/OH/AuNPs/aptamer in different concentrations of RIV (500, 300, 200, 100, 50, 20, and 10 nM); (**B**) calibration curve of the aptasensor. Scan rate is 100 mVs^−1^. Supporting electrolyte is [Fe(CN)_6_]^3−/4−^/KCl (0.01 M). (Incubation time is 20 min). (E_beging_ = −1 V, E_end_ = 1 V, amplitude time = 0.25 s, frequency 1 Hz). (n = 3).

The analytic performance of the generated aptasensor was investigated using the SWV method in 0.01 M [Fe(CN)_6_]^3−/4−^ solution under optimized conditions in a standard sample. The relative electrochemical signal increased gradually upon the enhancement of the RIV concentration and an excellent linear relationship between the current response signal and the RIV concentration was achieved. The calibration curve was linear over a concentration range of 10–500 nM, with a regression equation of (I/μA) = −0.4058 C (RIV) + 276.88 and R^2^ = 0.9917. The signal-to-noise ratios (S/N) of 3 and 10 were used to calculate the LOD and LOQ, respectively. The LOD and LOQ values were found to be 6.6 and 21.9 nM, respectively (Figure 2A,B).

### 3.8. Comparison of the Method with Other Procedures

In Table 1, the analytical performances of various methods, in the last 10 years, for detection of RIV including high-performance liquid chromatography (HPLC) with various detectors such as UV, photodiode array (PDA), mass spectroscopy, or tandem mass methods have been compared.

The majority of the reported HPLC techniques have various flaws, including low sensitivity, complicated mobile phases, and time-consuming extraction procedures with restricted applicability, as illustrated in Table 1.

Regarding Table 1, Cini and coworkers focused on the follow-up concentrations of RIV in samples taken from treated individuals [80]. According to this study, suitable analytical results were obtained. Additionally, for the simultaneous measurement of RIV in human plasma, Gouveia and his research team used a high-performance liquid chromatography–diode array detector (HPLC-DAD) method [81]. Foerster et al. employed the normal-phase LC system in LC-MS/MS for the measurement of RIV concentrations in clinically specific conditions [82].

Prior research indicates that the sensitivity of HPLC-UV and LC-MS/MS in the examination of RIV is nearly equal (Table 1) despite the invention and validation of several LC-MS methods specifically for the measurement of RIV.

As we know, these methods need to be run by an expert operator. Additionally, in HPLC-based analytical techniques, samples need to go through pretreatment steps. As such, samples should be prepared for inquiry according to the appropriate the pretreatment steps, including extraction, concentration, dilution, clean-up, and derivatization. For this purpose, solid-phase extraction (SPE), protein precipitation (PP), and liquid–liquid extraction (LLE) are often considered [83,84].

To overcome these limitations, Zhao et al. used simple protein precipitation for plasma sample preparation in the quantification of RIV. However, the Thermo Scientific^®^ Turbo Flow^®^ technology used in this assay was unusual and is not found in most laboratories [85]. Another test utilized tandem mass spectrometry to detect RIV in human plasma samples. However, the low LLOQ (23 ng/mL) showed that it is insufficiently sensitive to estimate concentrations [86].

Similarly, Wiesen and Foerster’s research groups used paramagnetic grains for SPE and applied it for the detection of RIV using tandem mass spectrometry. However, this method was time-consuming and expensive [32,82].

Although, HPLC-based methods have low limits of detection (LODs), they have significant limitations, including being time-consuming, sample pre-treatment procedures, difficult processes, high costs, long processing times, complicated manipulation, and the need for experienced operating personnel as well as advanced instruments. So, the previous analytical results obtained from monitoring RIV in clinical samples show that there is no established methodology that uses an electrochemical detector for the quantification of RIV with HPLC analysis or an optical and electrochemical biosensor for RIV assessment in real samples.

To address these issues, our research team proposed a simple, quick, and commonly utilized approach for quantifying RIV using an electrochemical-based aptasensor and that integrates biotechnology with electronic systems for the efficient monitoring of RIV in real samples.

A comparison of the results obtained in this study with previously reported works (Table 1) shows that the developed method shows some advantages over previous approaches, which are related to the high surface area of the ITO-PET-OH-AuNPs, the appropriate dense loading of the aptamer on the surfaces of electrodes, and the strong binding of aptamer-Au(S-Au) to the aptasensor. Additionally, ITO-PET-OH-AuNPs-aptamer is a suitable platform for the detection of RIV in biological fluids. The specific binding of aptamer-RIV can be loaded for the selective recognition of the drug in complex matrices. As a consequence, the engineered aptasensor was able to detect RIV with great accuracy.

RIV was identified using the ITO-PET-OH-AuNPs-aptamer matrix, which had excellent stability, suitable surface area, and feasible biological activity.

Strong points of our study include the use of the electrochemical aptasensor procedure for the determination of RIV in EBC for the first time.

It is important to point out that the quantification of RIV in EBC in biological samples carried out in this work represents a simple and non-invasive sampling method for therapeutic drug monitoring (TDM) applications.

We believe that the designed approach is a good bio-assay for determining RIV quantitatively in real samples.

In terms of response, the proposed aptasensor approach clearly outperforms previously reported methods. Indeed, because of their advantages, such as simple instrumentation, rapidness, excellent compatibility with miniaturized technologies, and their remarkable sensitivity to the target, electrochemical aptasensor-based methods have more applications compared to other sensing technologies [56,57].

As such, electrochemical-based aptasensors may be utilized as an alternative to tedious analytical procedures, particularly in low-income nations due to their simplicity, affordability, and cost-effectiveness. This aptasensor can also be used as a novel biodevice in clinical analysis.

### 3.9. RIV Measurement in the Plasma and EBC Samples

To assess the ability of the proposed aptasensor to detect RIV in biological samples, the performance of the sensor to measure RIV in human plasma and EBC samples was investigated using the ChAs technique and [Fe(CN)_6_]^3−^/^4−^/KCl (0.01 M)] as the supporting electrolyte. For this purpose, 5 µL of unprocessed human plasma and EBC samples were mixed with various amounts of RIV and were incubated on the surface of the ITO-PET-OH-AuNPs-aptamer.

**Figure 3:**
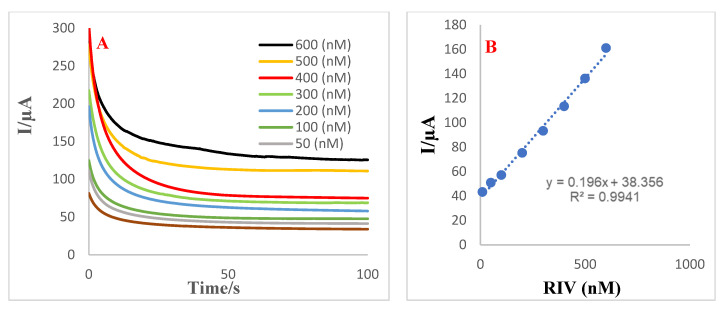
(**A**) ChAs of the engineered aptasensor in different concentrations of Riv (50 nM to 600 nM) in unprocessed human plasma samples. (**B**) Relationship between I_pa_ and Riv concentration in plasma sample. t_eq_ = 2, where E_being_ = −1 V, E_end_ = 1 V, E_step_ = 0.1 V, amplitude = 0.001 V, and frequency = 10 Hz. Using [Fe(CN)_6_]^3−/4−^/KCl (0.01 M) as the supporting electrolyte (n = 3).

**Figure 4:**
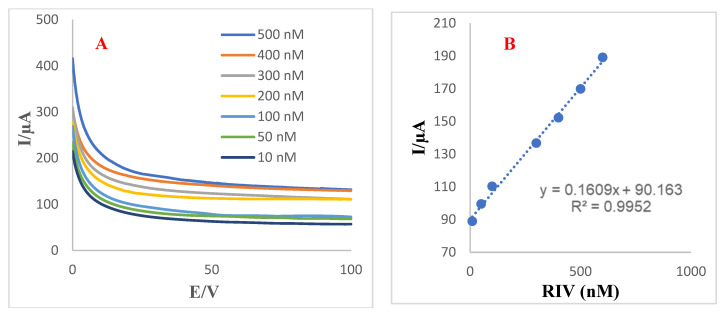
(**A**) ChAs of the engineered aptasensor in different concentrations of Riv in unprocessed human EBC samples. (**B**) Relationship between Ipa and Riv concentration in plasma sample. t_eq_ = 2, E_being_ = −1 V, E_end_ = 1 V, E_step_ = 0.1 V, amplitude = 0.001 V, and frequency = 10 Hz. Using [Fe(CN)_6_]^3−/4−^/KCl (0.01 M) as the supporting electrolyte (n = 3).

The calibration curves were then obtained by plotting the peak current versus various concentrations of RIV that had spiked in the blank human plasma and EBC samples (Figure 3 and Figure 4) under optimum conditions. The characteristics of the proposed sensor for RIV determination in human plasma and EBC, including the LOD and LOQ values as well as the precision (expressed as relative standard deviations, RSD %) and recovery study results, have been summarized in Table S1. The recovery of the added RIV resulted in recovery rates from 96.2 to 108.1% and from 88.7 to 104.3% for plasma and EBC, respectively, which were within the required limit of 80.0–120.0%, which is indicative of the accuracy of the aptasensor in the measurement of RIV [90]. The RSD values for plasma and EBC were calculated as 1.6–5.0% and 0.4–6.1%, respectively (Table S2), which are much lower than 20.0%. Therefore, the developed aptasensor-based method can be considered precise [90].

The spiked samples were also analyzed by the LC-MS/MS method as a positive control assay, and the results are shown in Table S3. As it can be seen, there is good agreement between the results obtained from the proposed sensor with those obtained from the LC-MS/MS method. The therapeutic level of RIV varies between 27.5 and 575.5 nM [91]. Bearing in mind that the (LODs) values for of the aptasensor developed for plasma and EBC in our study were 14.08 and 6.03 nM, respectively, it seems that the proposed sensor has a good potential to measure RIV in the clinical samples.

### 3.10. Analytical Method Validation

#### 3.10.1. Selectivity of the Proposed Aptasensor

Undoubtedly, one of the most striking properties of a biosensor is the ability to measure the targets in a sample containing similar species as interfering compounds. Multiple potentially interfering species in real samples that may interfere in the determination of RIV, such as ascorbic acid (AA), arginine (Arg), glucose (GlC), L-cysteine (L-Cys), glycine (Gly), tyrosine (Tyr), uric acid (UA), glutamic acid (Glu), magnesium (Mg), and L-proline (Pro), were prepared in standard solution to evaluate the aptasensor’s selectivity in the supporting electrolyte (Figure S11A (see Supporting Information)). To achieve this, the SWV technique was used. The selectivity of the ITO-PET-OH-AuNPs-aptamer electrode was evaluated in different types of interfering compounds in the supporting electrolyte [Fe(CN)_6_]^3−/4−^/KCl (0.01 M)]. As illustrated in Figure S14B, in this system, the highest current intensity is related to RIV, which is about 420 µA. This indicates a high affinity binding between the aptamer and RIV. L-Tyr may cause more interference than other species because due to its close resemblance to RIV, it has a higher IP than the other species. Additionally, the electrochemical aptasensor showed a higher response to Glu, AA, UA, and Tyr than other interfering compounds, but the responses were lower than RIV. This suggests that these species have similar structures with the candidate drug and can bind to aptamer. Other species such as Arg, Cys, and Pro show much weaker affinities to the aptamer, and as a result, they will not interfere with the system.

It is important to emphasize that the constructed aptasensor did not show significant responses to other tested compounds. Whereas L-Gly, Mg^2+^, and glucose have the lowest current intensity among the selected species, this indicates that they have no interaction with the desired aptamer, which may be due to their small size. So, the coexistence of these species certainly does not cause interference with RIV detection.

Therefore, adding interfering substances resulted in no discernible increase in the peak current, and the interference was nearly negligible, indicating that the fabricated aptasensor exhibits remarkable specificity for the recognition of RIV.

To test the specificity of the electrochemical aptasensor towards RIV, the ability of ITO-PET-OH-AuNPs-aptamer to detect RIV was evaluated in the presence of drugs (hydrochlorothiazide, metoprolol, nitroglycerin, aspirin, and losartan) that could be co-administrated with RIV. The drugs were prepared in the standard solution and were evaluated in the supporting electrolyte. The anti-interference performance of the sensor was evaluated by measuring the RIV peak current value in the presence of the selected drugs and comparing it to that of the RIV response in the absence of the drugs. A paired *t*-test was performed to check the significance of the data using SPSS Statistics version 20.0 software. With all of the tested drugs, apart from aspirin (*p* = 0.042), no statistically significant differences (*p* < 0.05) were obtained between the responses (Figure S15B). This confirms the high specificity of the biosensor towards RIV in the presence of the selected drugs that could be co-administrated with RIV, with no type II errors.

#### 3.10.2. Stability of the Engineered Aptasensor

Stability is the ability to efficiently reuse a sensor and is a prominent factor in the evaluation of engineered aptasensor performance. Indeed, the aim of the stability testing method is to determine whether or not the analyte is stable with changes in the environment, such as changes in the matrix and storage conditions. For this purpose, the aptasensor’s stability in relation to the number of cycles was assessed by monitoring the drop in the peak current intensity during repeated CV using an ITO PET-OH-AuNPs electrode.

Figure S16 shows the CVs of the ITO PET-OH-AuNPs electrode with different cycle numbers in the supporting electrolyte [Fe(CN)_6_]^3−/4−^/KCl (0.01 M)] solution. The results indicated that there was little substantial difference between the first cycle, the fifth cycle, and the tenth cycle because the peak currents of the aptasensor retained high stability when the number of cycles increased to 10 and then decreased very smoothly. However, with a rise in the number of cycles to 50, the peak current decreased intensively. This indicates that after 50 cycles, the electrode surface is practically inactive and confirms the instability of the interface. According to these findings, the related electrode has excellent performance and repeatability for up to ten cycles, and the engineered substrate can be used for up to ten cycles.

#### 3.10.3. Intra-Day Repeatability

For practical applications, the stability of the engineered aptasensor is crucial. The intra-day stability of the electrode was studied using one electrode by putting it in the fridge for three consecutive days and recording the CVs. The electrochemical behavior of the developed aptasensor was studied by recording the CVs in the supporting electrolyte [Fe(CN)_6_]^3−/4−^/KCl (0.01 M)] solution. According to Figure S17B, the electrode’s peak current intensity was 482.96 on the first day and decreased to 201.17 on the second. However, on the third day, the electrode had practically no current intensity, and it had reached almost zero. This result indicates that the prepared electrodes are not stable on different days because there is a big difference between the first-day current and the currents on the other days, and after 72 h they completely lose their function. The reason for this is the low stability of the substate after 3 days. Therefore, this sensor has poor stability and can only be used once. The repeatability results of electrochemical aptasensor are included in Table S4.

## 4. Conclusions

Using an affinity chromatography-based SELEX strategy, we have identified, developed, and characterized a novel aptamer that can bind to RIV with a high level of affinity and specificity for the first time. After that, the RIV-specific aptamer was used to develop and fabricate a label-free electrochemical nano-aptasensor for RIV detection in human plasma and EBC samples with LOD values of 14.08 and 6.03 nM, respectively, and a wide linear range of 10 nM to 500 nM. RIV determination was carried out under the optimized conditions. Therefore, the proposed aptasensor is able to quantify the RIV in human plasma and EBC as biological samples with a low LOD, a wide linear detection range, good selectivity, and high stability. Taking into account the therapeutic levels of RIV, which lie between 27.5 and 575.5 nM [91], this sensor shows good potential for clinical applications.

## Figures and Tables

**Table 1:** Analytical figures of merit of previously developed methods toward determination of RIV in biological samples.

Method	Biological Matrix	LOD/LOQ/LLOQ	Linear RANG	Ref
HPLC-UV	Human plasma	11.6 nM (LOD)	11.6 to 34.8 nM	[80]
HPLC-DAD	Human plasma	0.0165 μg/mL (LLOQ)	0.017 to 5.28 μ/mL	[81]
(UPLC/MS/MS)	Human plasma	2.3 nM (LLOQ)	2.3 to 2325 nM	[82]
UPLC/MS/MS	Human plasma	2.3 (LLOQ)	2.3 to 2325 nM	[83]
SALDI-MS	Human urine and serum	Urine: 6 nM (LOD) Serum: 60 nM (LOD)	5 to 500 nM in plasma	[84]
UHPLC-MS/MS	Human plasma and breast milk	10.4 nM in plasma (LLOQ) 11.16 nM in breast milk (LLOQ)	11.6 to 1163nM in plasma 11.6 to 581.4 nM in breast milk	[85]
UPLC-MS/MS	Human plasma	55.8 nM (LOD)	39.3 to 465.8 nM	[86]
UPLC/MS/MS	Human plasma	5.8 nM (LLOQ)	5.8 to 1744.1 nM	[82]
UPLC/MS/MS	Human plasma	1.14 nM (LOD) 3.42 nM (LOQ)	4.65 to 1162.7 nM	[32]
LC-MS/MS	Human plasma	55.8 nM (LLOQ)	55.81 to 2232.5 nM	[38]
SPE–HPLC–UV	Human plasma	11.6 nM (LOD)	23.25 to 9302 nM	[87]
LC-MS (High resolution)	Human plasma	2.3 nM (LLOQ)	2.3 to 1162.7 nM	[88]
UPLC-MS/MS	Human plasma	1.25 nM (LLOQ)	1.86 to 1860.4 nM	[89]
Electrochemical aptamer biosensor	Human plasma and EBC	6.03 nM in EBC (LOD) 14.08 nM in plasma (LOD)	10 to 600 nM in plasma and EBC	This Study
LCMS/MS	Human plasma and EBC	0.5 nM in EBC (LOD) 1.1 nM in plasma (LOD)	10 to 500 nM in plasma and EBC	

Abbreviations: HPLC, high-performance liquid chromatography; LOD: limit of detection; LLOQ, low limit of quantification; LC-MS/MS, high-performance liquid chromatography–mass spectroscopy/mass spectroscopy; UPLC-MS/MS, ultra-performance liquid chromatography–tandem mass spectrometry.

## Data Availability

Not applicable.

## Supplementary Materials

The following supporting information can be downloaded at: https://www.mdpi.com/article/10.3390/bios12100773/s1, **Figure S1.** Chemical structure of Rivaroxaban. **Figure S2.** Coupling reaction of RIV with epoxy-activated Sepharose bead. **Figure S3.** The FT-IR spectra of A, RIV, B, RIV-acidic derivative. **Figure S4.** ^1^H NMR spectra of compound (1). **Figure S5.** FT-IR spectrum of A, RIV acidic derivative, **B**, RIV-hexandiamine-Sepharose, **C**, hexandiamine-Sepharose, **D**, epoxy-Sepharose. **Figure S6.** FE-SEM images of Sepharose beads at different magnification. For first row: epoxy-activated Sepharose 6B, second row: Sepharose-hexanediamine, and third row: Riv-(Sepharose-hexanediamine) matrix. **Figure S7.** SPR sensor response for determination of the dissociation constants (kd) of thiolated aptamer in combination with a concentration series of the target RIV (20–500 nM). On the basis of the obtained curves, non-linear regression analysis was used to compute Kd values. **Fig****ure** **S8.** ChA of electrodeposition of AuNPs on the surface of ITO-PET-OH at the potential of E = 0.0 V, time = 200 s. 0.01 M HAuCl_4_·3H_2_O and 0.01 M CTAB and arginine (0.003 g). **Figure S9.** (**A**) CVs of ITO-PET/OH/AuNPs in 0.01 M Fe(CN)_6_]^3−/4−^ containing KCl, in various potential sweep rates (from inner to outer): 10, 50, 100, 150, 200, and 1000 mVs^1^, respectively. (**B**) Dependence of anodic/cathodic peak currents vs. sweep rate. (**C**) Dependence of anodic/cathodic peak currents vs. square root of potential sweep rate. (**E**) Tafel plot (variation of peak potential versus *v*n I_pa_. (**F**) Dependency of *v*ln I_pa_ versus ln*v*. **Figure S10A**. FESEM images ITO PET-OH electrode in different magnifications. **Figure S10B**. FE-SEM images of ITO PET-OH modified Au electrode at various magnifications. **Figure S10C**. FE-SEM of ITO PET-OH-AuNPs-aptamer in different magnifications. **Figure S10D**. FE-SEM image of ITO PET- OH-AuNPs-aptamer after incubation with RIV in different magnification. **Figure S11.** Topographical AFM images of (**A**) ITO film on PET, (**B**) ITO-PET/OH, (**C**) ITO-PET/OH/AuNPs, (**D**) electrode substrates after thiol aptamer assembly and planar ITO-PET/OH/AuNPs/aptamer/RIV substrates. **Figure S12.** FT-IR spectra of (**A**) Bare ITO-PET, (**B**) ITO-PET-OH-AuNPs, and (**C**) ITO-PET-OH-AuNPs-aptamer-RIV, respectively. **Figure S13**. Optimization of various incubation time (20, 40, 60, 80, 100,120 and 360 min) for aptamer immobilization (A) CVs of ITO PET-OH-Au NPs-aptamer in ferrocyanide/ferricyanide/KCl (0.01 M) at the potential rang of −1 to +1 V, sweep rate: 100 mv/s (B) Histogram of peak current versus time of incubation. (n=3). **Figure S14.** (**A**) SWV of aptasensor in the presence of different interfering agents. (**B**) Variation of peak currents versus type of interfering species. Supporting electrolyte is 0.01 M [Fe(CN)_6_]^3^^−^^/4^^−^, SWV parameters: potential range of −1 V to +1 V with a step potential of 0.01 mV, amplitude 0.25 mV and frequency 1 Hz. (n = 3). **Figure S15**. (**A**) SWV of aptasensor in the presence of different species (hydrochlorothiazide, metoprolol, nitroglycerin, atorvastatin, aspirin, and losartan). (B) Dependency of peak currents of aptasensor versus the type of interfering agents. The concentrations of analogs were 1 mM. Supporting electrolyte is 0.01 M [Fe(CN)6]^3−/4−^, SWV parameters: potential range of −1 V to +1 V with a step potential of 0.01 mV, amplitude 0.25 mV, and frequency 1 Hz. Statistical significance was determined by Paired *t*-test. The aspirin shows a significantly lower response than other drugs (*p* < 0.05) indicating significant differences relative to displayed bars as determined. (n = 3). **Figure S16.** (A) CVs of ITO-PET-OH-AuNPs in different cycle number (1–50 Cycle). (**B)** Histogram of peak current versus number of cycle. Sweep rate is 100 mV/s. supporting electrode is [Fe(CN)_6_]^3−/4−^/KCl (0.01 M)]. (n = 3). **Figure S17.** (A,B) CVs of ITO-PET-OH-AuNPs/Aptamer in the various storage time (24, 48, and 72 h) in supporting electrolyte [Fe(CN)_6_]^3−/4−^/KCl (0.01 M)] sweep rate is 100 mv/s. (1–3 days). (n = 3). **Scheme S1**. A schematic outline of the SELEX procedure using modified RIV-Sepharose toward aptamer discovery. **Table S1.** Validation data of proposed method for quantification of RIV in exhaled breath condensate (EBC) and human plasma samples (n = 3). **Table S2**. Recovery of RIV from exhaled breath condensate (EBC) and plasma samples (n = 3). Data are presented as mean ± relative standard deviation (RSD). **Table S3**. Comparison of the recovery of RIV from exhaled breath condensate (EBC) and plasma samples (n = 3), and the relative standard deviations (RSD %) analyzed by the proposed aptasensor and LC-MS/MS method. **Table S4**. The repeatability of aptasensor.
