# Supplementary material for: Selection of Specific Aptamer against Rivaroxaban and Utilization for Label-Free Electrochemical Aptasensing Using Gold Nanoparticles: First Announcement and Application for Clinical Sample Analysis
Source: Biosensors (Basel). 2022 Sep 20;12(10):773. doi: 10.3390/bios12100773 (PMC9599351; doi:10.3390/bios12100773)
Supplement: Supplementary file 1 [file biosensors-12-00773-s001.zip › biosensors-1911476-supplementary.pdf]

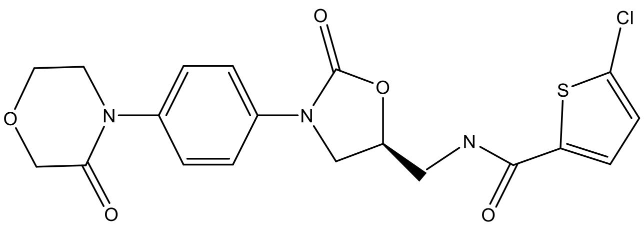

Figure S1. Chemical structure of Rivaroxaban

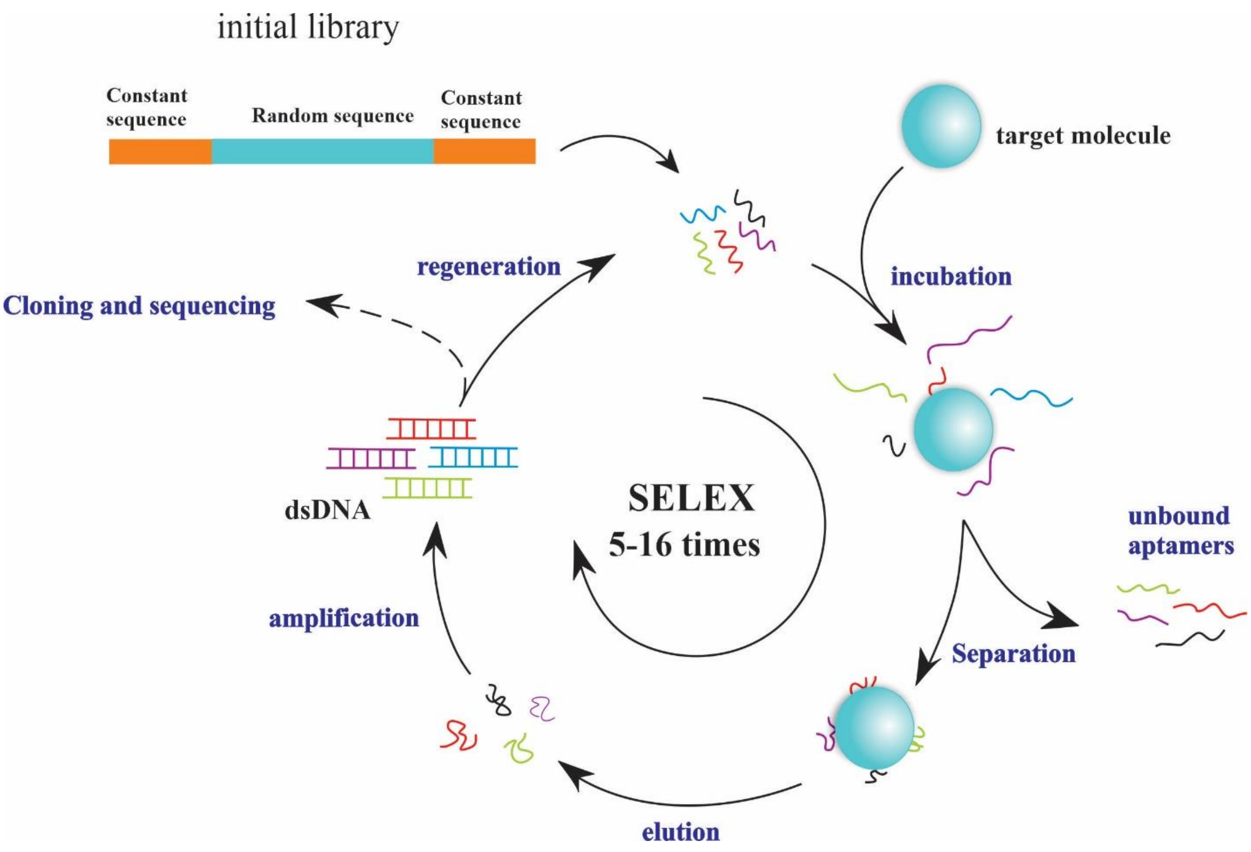

Scheme S1. A schematic outline of the SELEX procedure using modified RIV-Sepharose toward aptamer discovery.

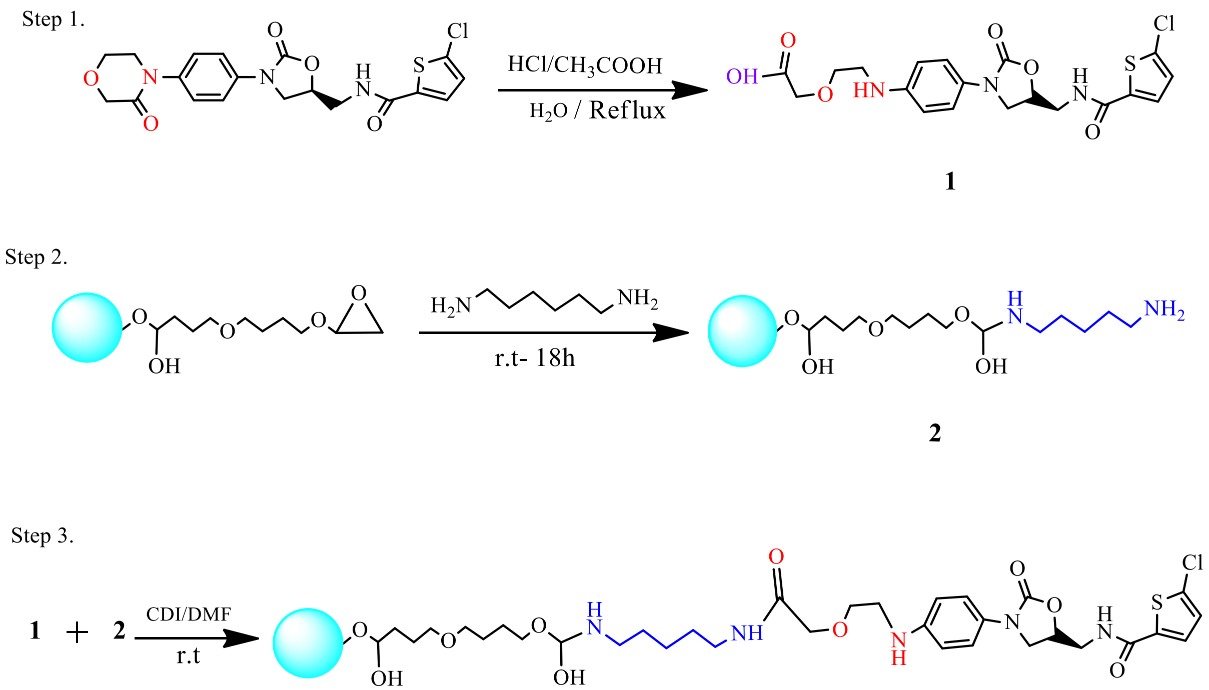

Figure S2. Coupling reaction of RIV with epoxy-activated Sepharose bead.

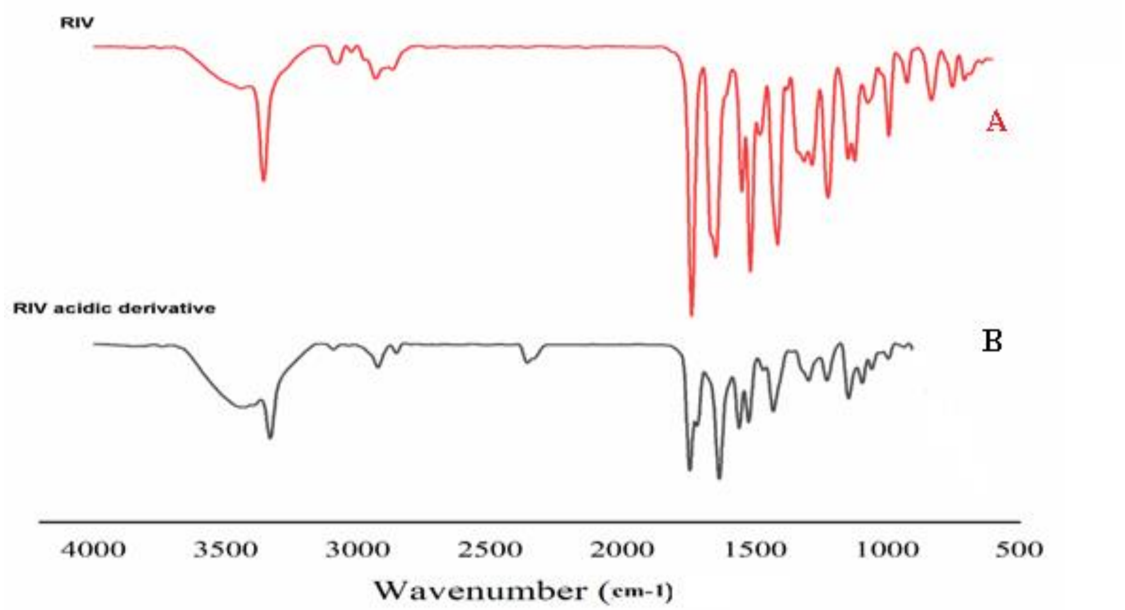

Figure S3. The FT-IR spectra of A, RIV, B, RIV-acidic derivative

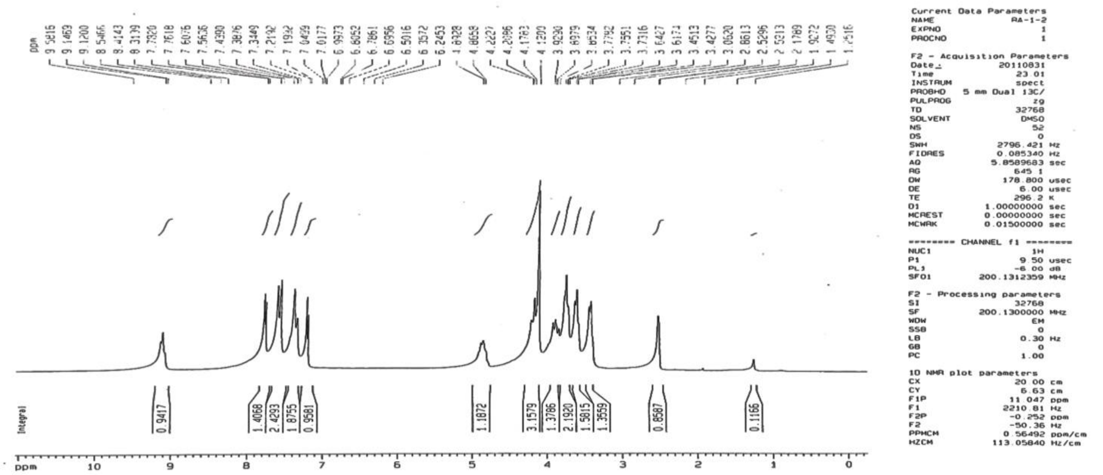

Figure S4. ^{1}H NMR spectra of compound (1)

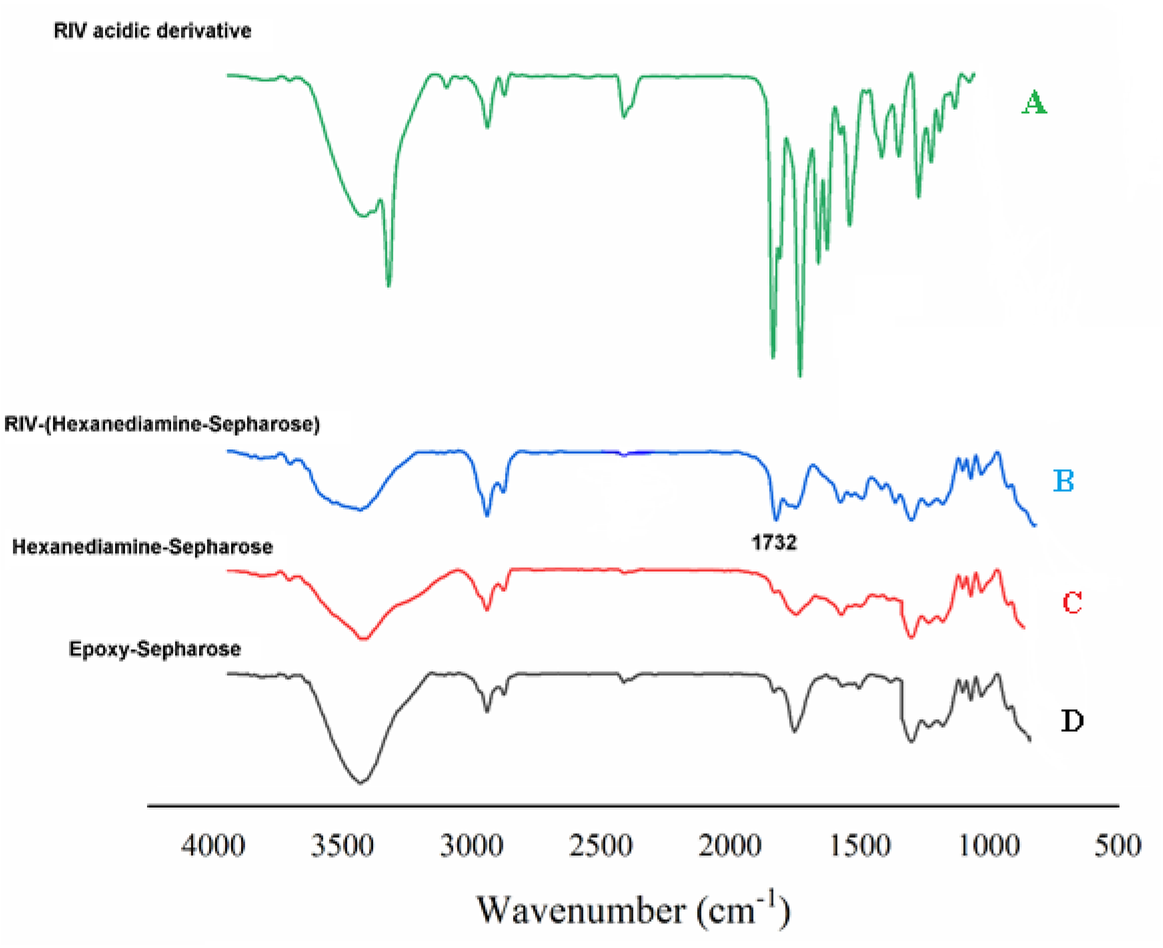

Figure S5. FT-IR spectrum of A, RIV acidic derivative, **B**, RIV-hexandiamine-Sepharose, **C**, hexandiamine-Sepharose, **D**, epoxy-Sepharose

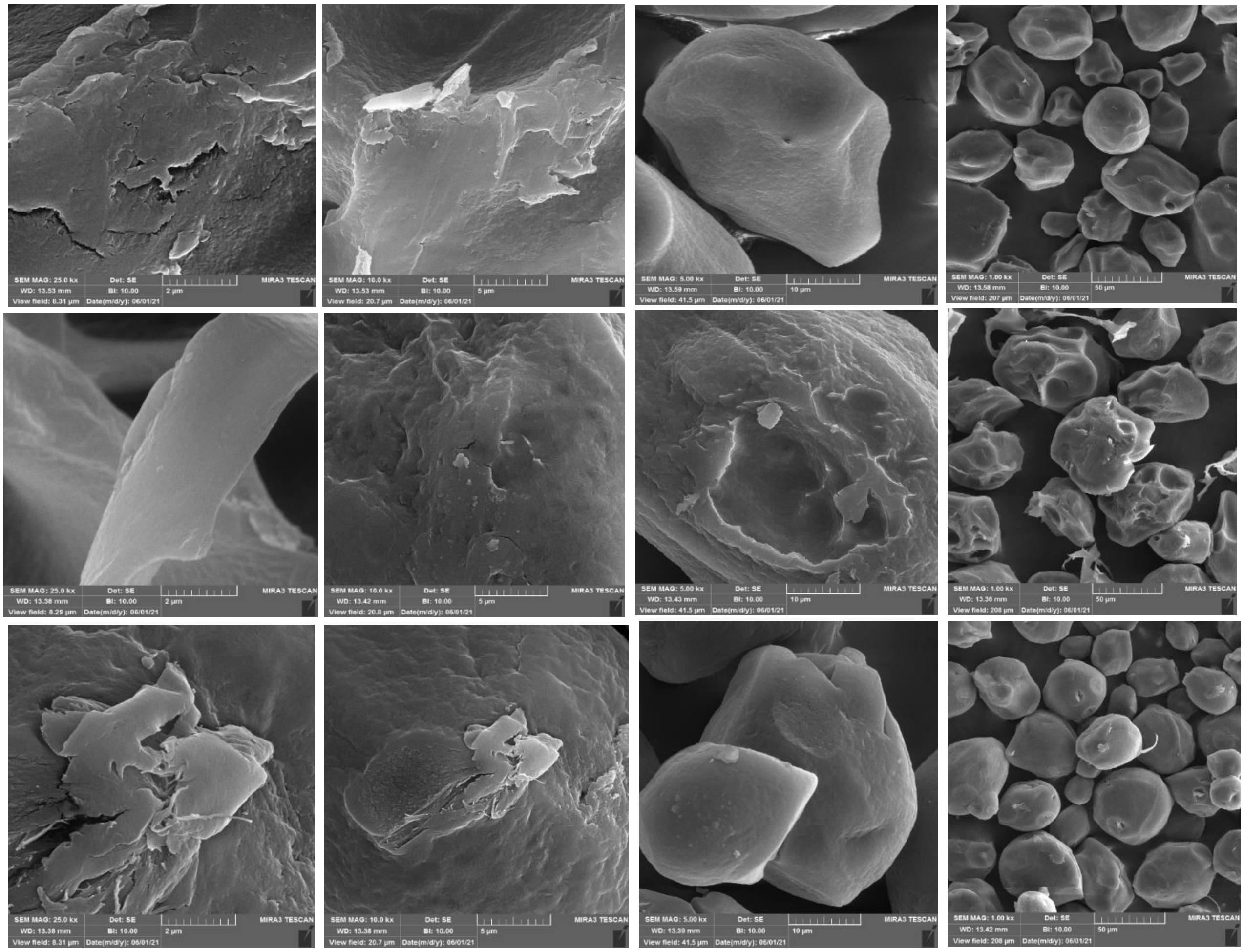

Figure S6. FE-SEM images of Sepharose beads at different magnification. For first row: epoxy-activated Sepharose 6B, second row: Sepharose-hexanediamine, and third row: Riv-(Sepharose-hexanediamine) matrix.

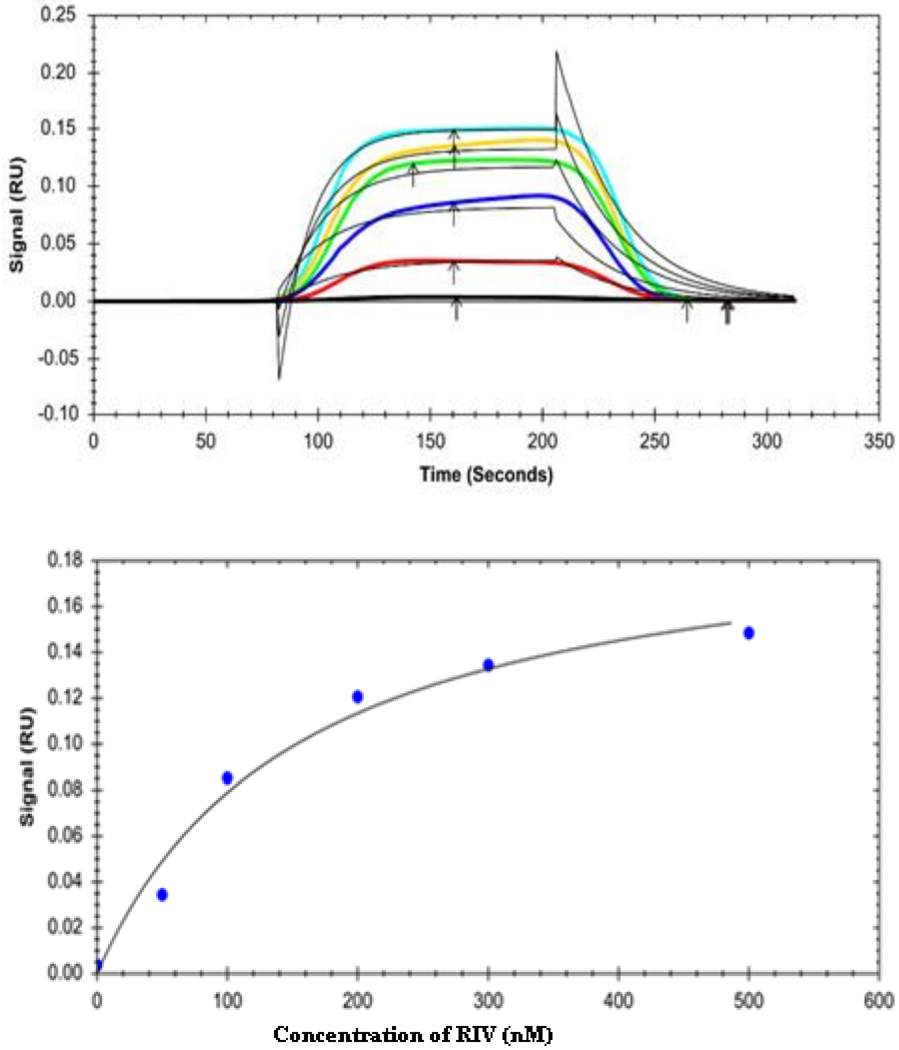

Figure S7. SPR sensor response for determination of the dissociation constants (kd) of thiolated aptamer in combination with a concentration series of the target RIV (20–500 nM). On the basis of the obtained curves, non-linear regression analysis was used to compute Kd values.

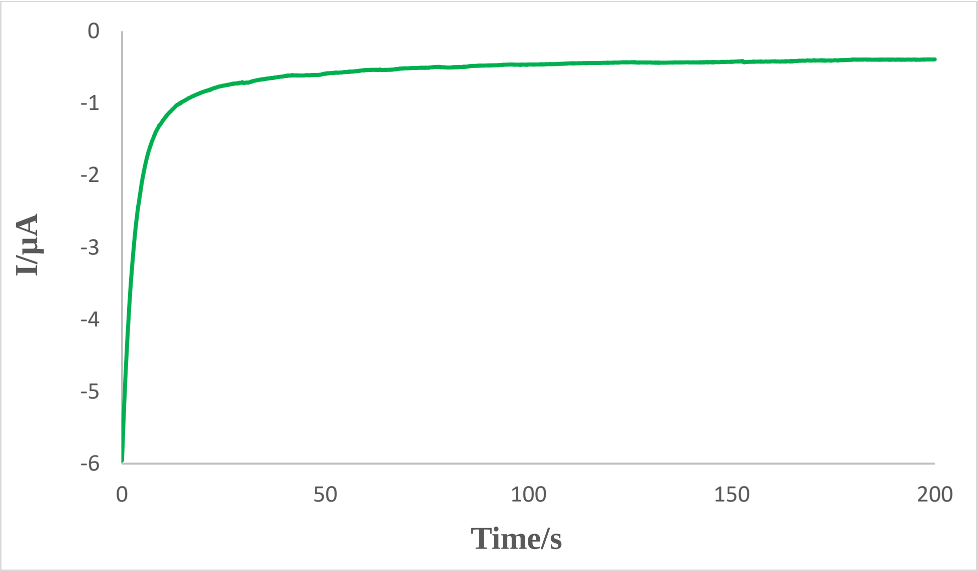

Figure S8. ChA of electrodeposition of AuNPs on the surface of ITO-PET-OH at the potential of E=0.0 V, time=200 s. 0.01 M $HAuCl_4 \cdot 3H_2O$ and 0.01 M CTAB and arginine (0.003 g).

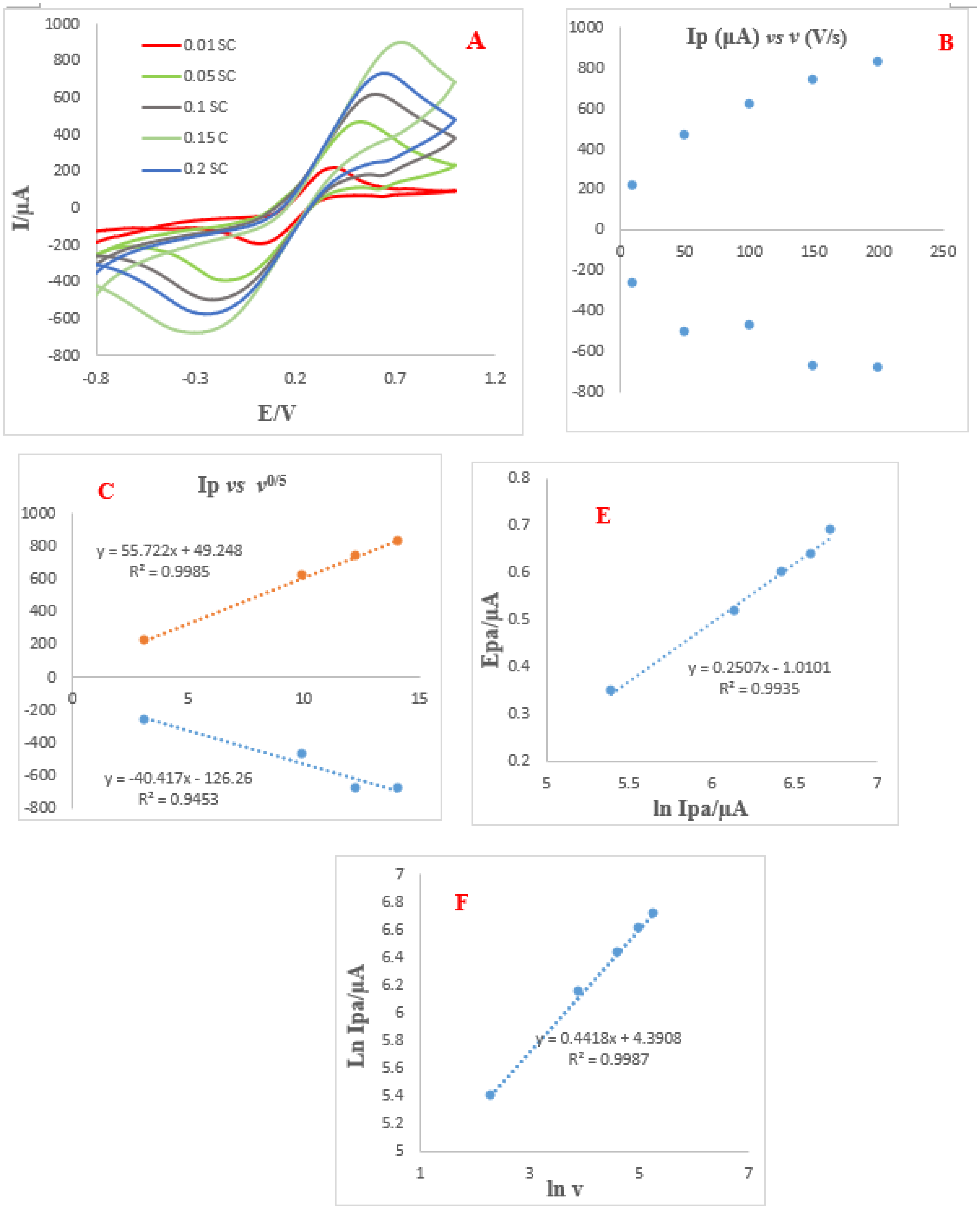

Figure S9. A) CVs of ITO-PET/OH/AuNPs in 0.01M $Fe(CN)_6]^{3-/4-}$ containing KCl, in various potential sweep rates (from inner to outer): 10, 50, 100, 150, 200, and 1000 mVs^{1}, respectively. **B**) Dependence of anodic/cathodic peak currents *vs.* sweep rate. **C**) Dependence of anodic/cathodic peak currents *vs.* square

root of potential sweep rate. **E**) Tafel plot (variation of peak potential *versus* vn I_{pa}. **F**) Dependency of $v$ln I_{pa} *versus* ln$v$.

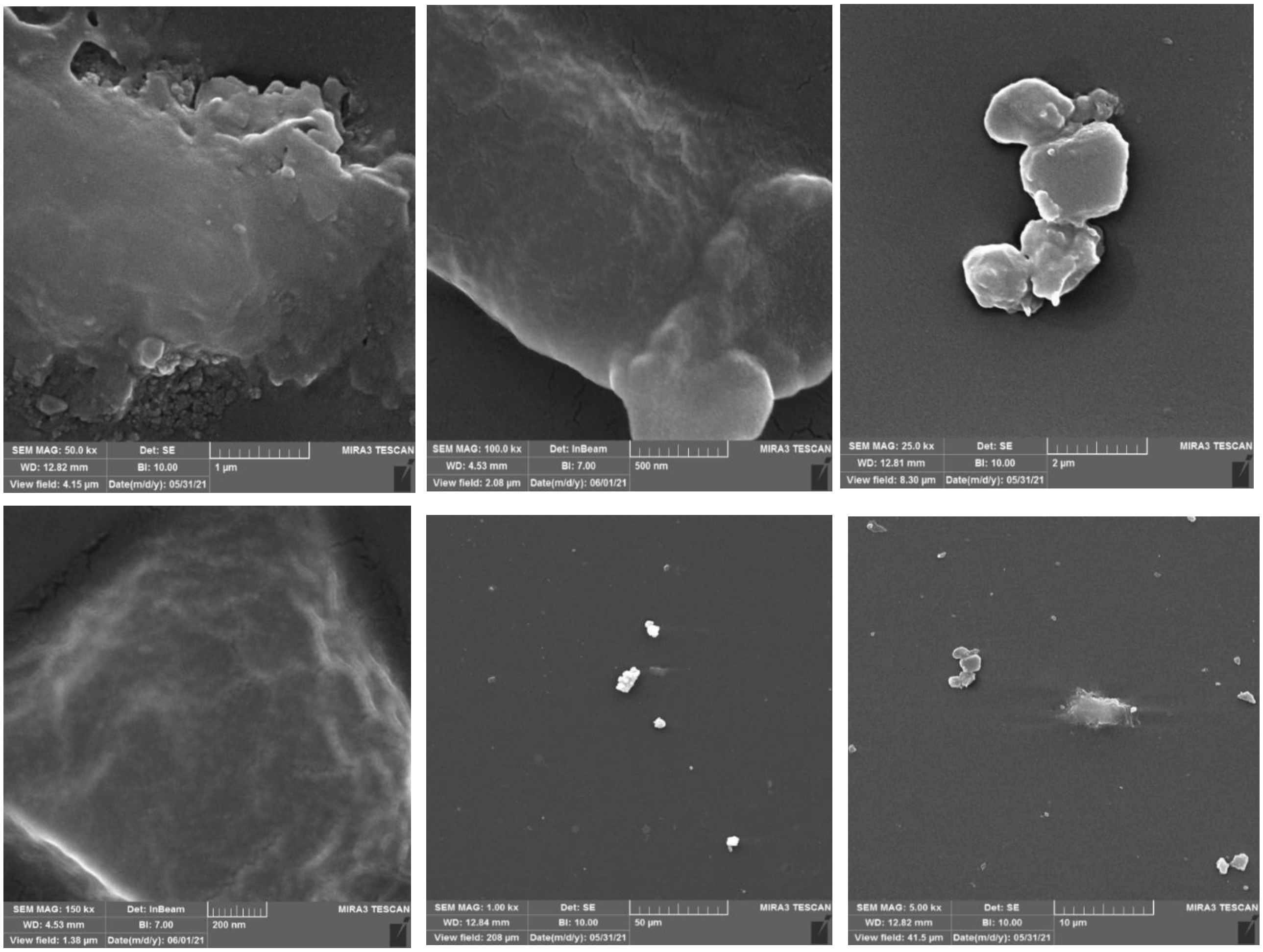

Figure S10A. FESEM images ITO PET-OH electrode in different magnifications.

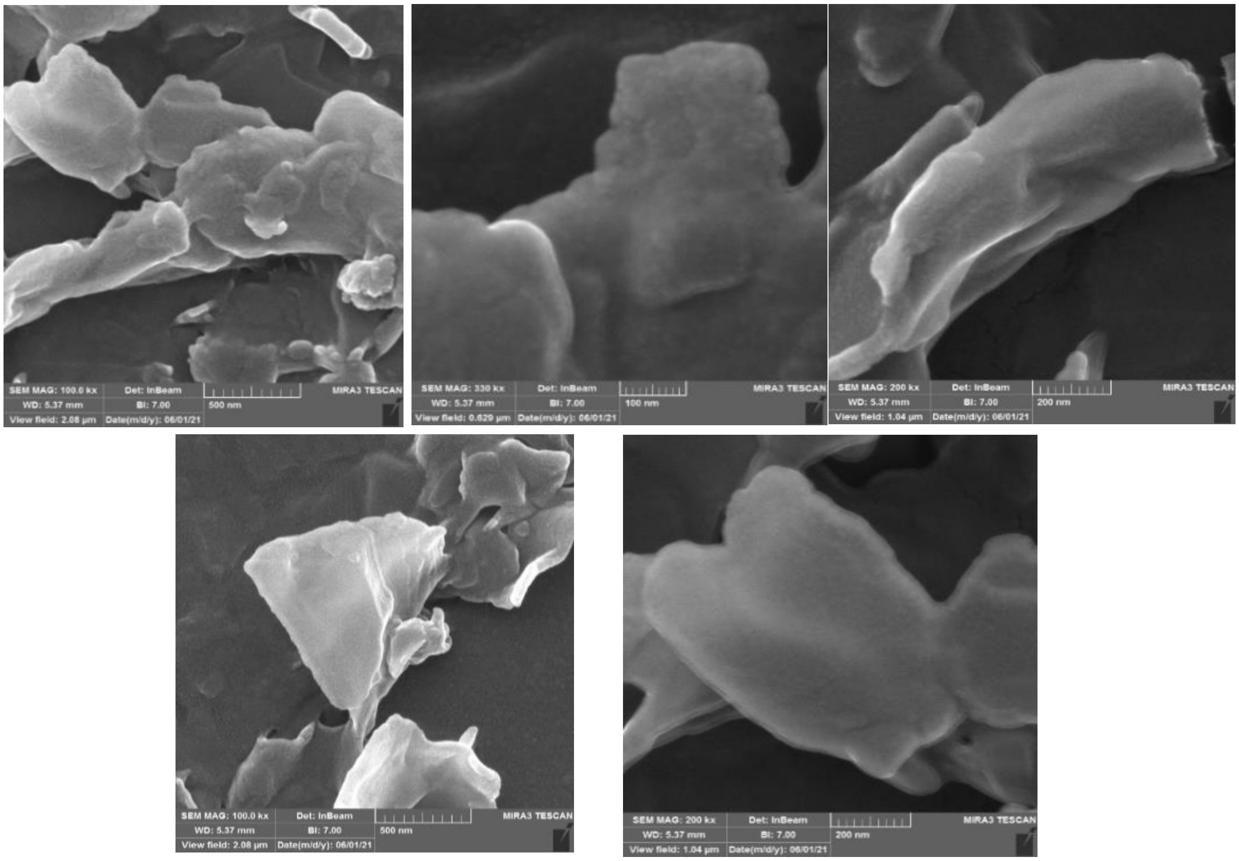

Figure S10B. FE-SEM images of ITO PET-OH modified Au electrode at various magnifications.

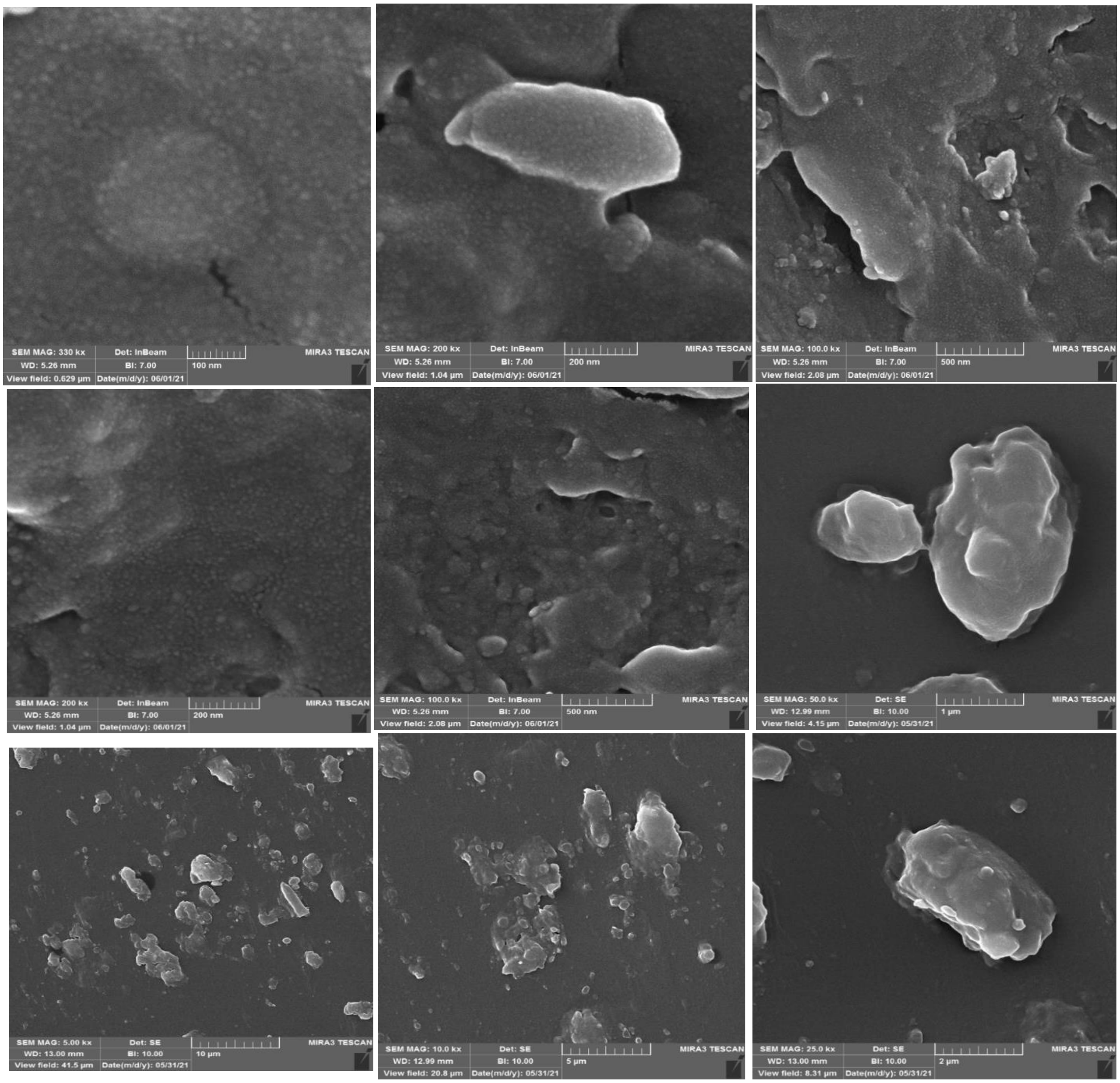

Figure S10C. FE-SEM of ITO PET-OH-AuNPs-aptamer in different magnifications.

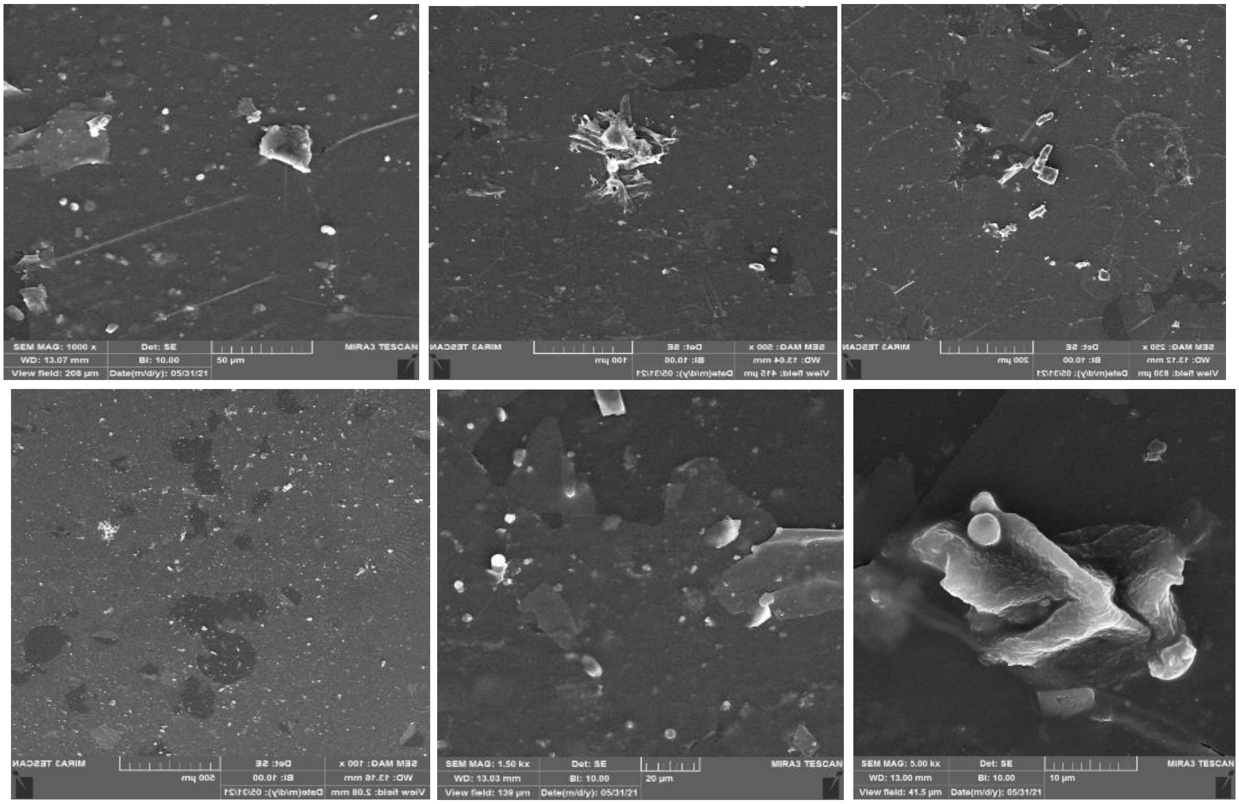

Figure S10D. FE-SEM image of ITO PET- OH-AuNPs-aptamer after incubation with RIV in different magnification.

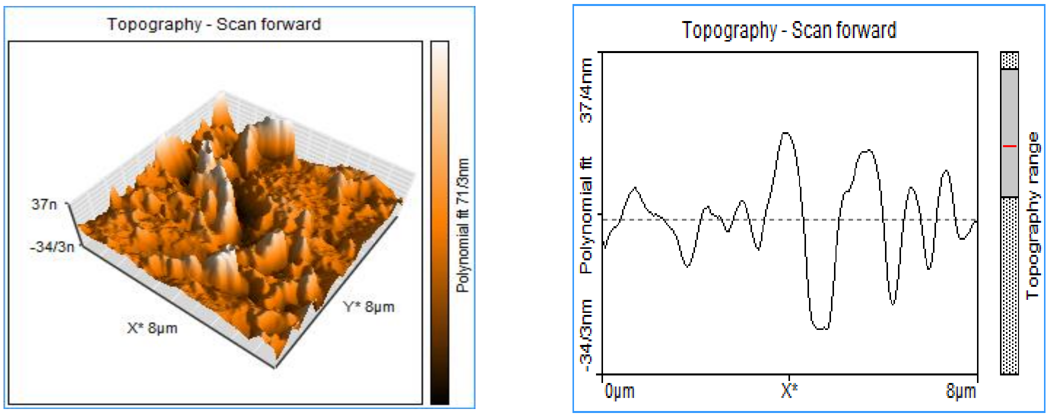

(**A**). ITO-PET-OH

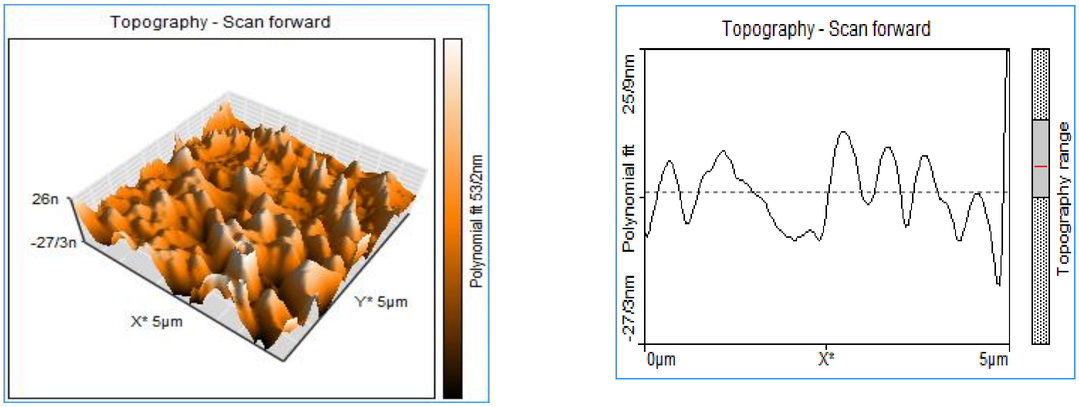

(**B**). ITO-PET-OH-AuNPs

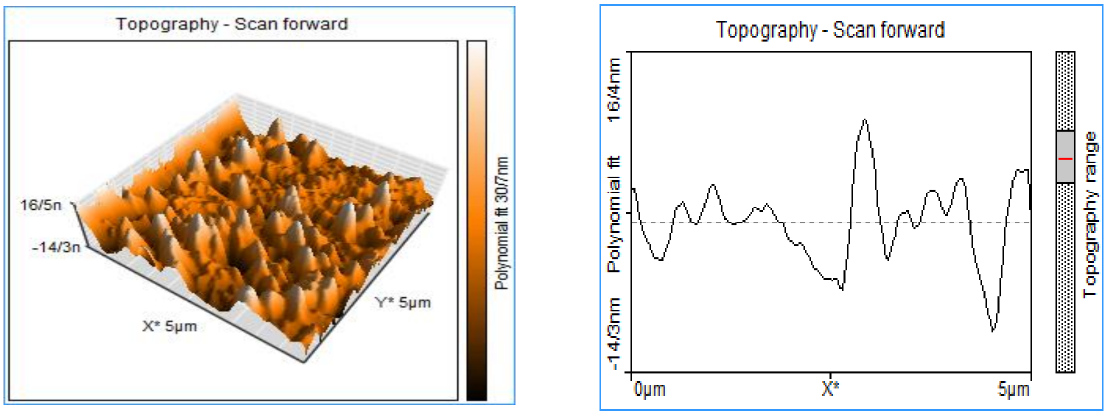

(**C**). ITO-PET-OH-AuNPs-aptamer

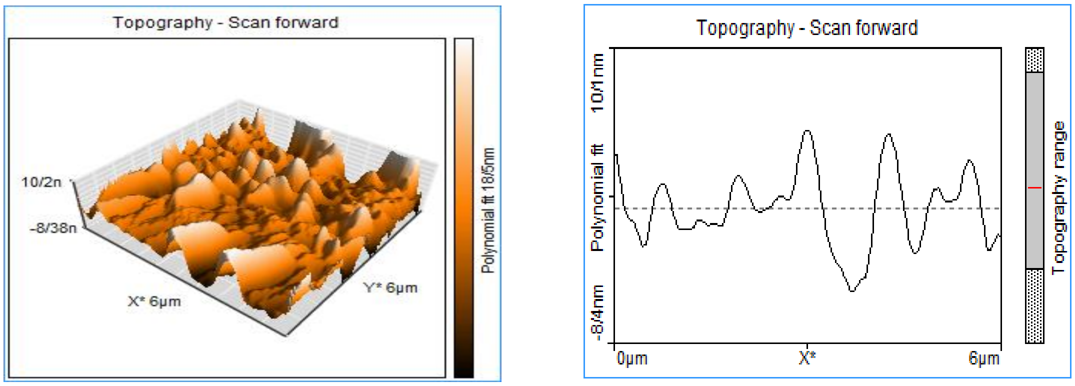

(**D**). ITO-PET-OH-AuNPs-aptamer/RIV

Figure S11. Topographical AFM images of (**A**) ITO film on PET, (**B**) ITO-PET/OH, (**C**) ITO-PET/OH/AuNPs, (**D**) electrode substrates after thiol aptamer assembly and planar ITO-PET/OH/AuNPs/aptamer/RIV substrates

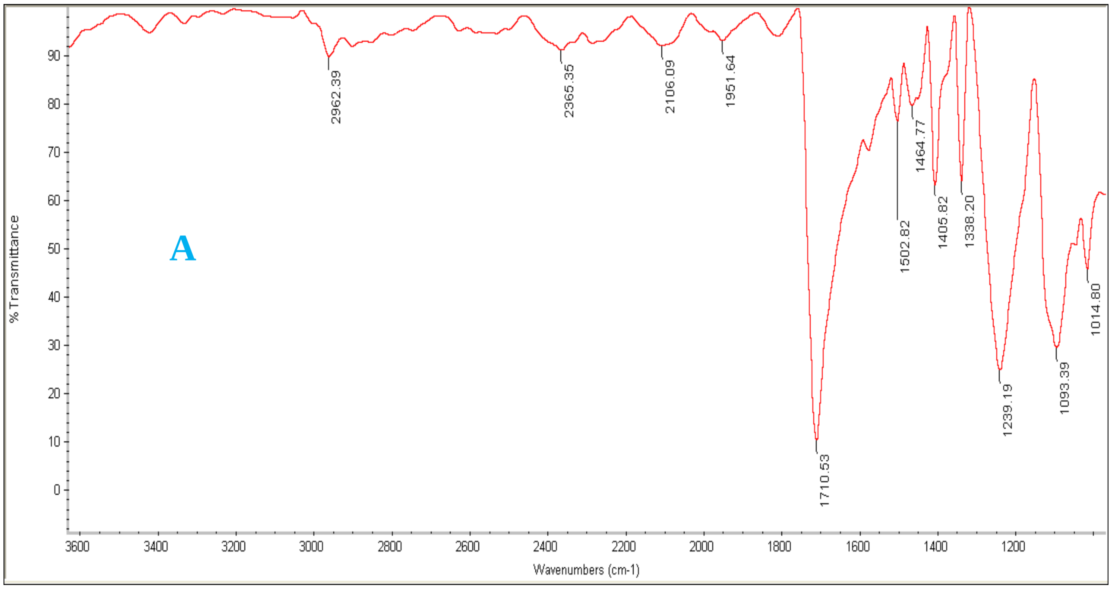

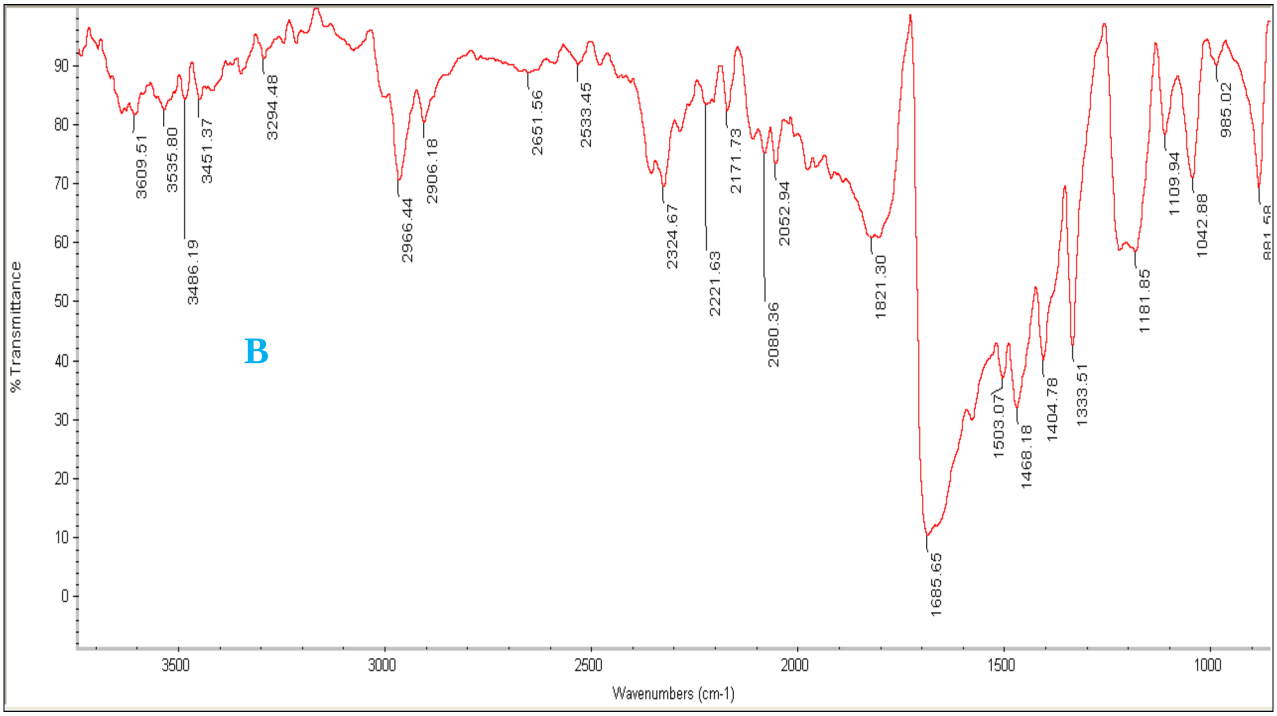

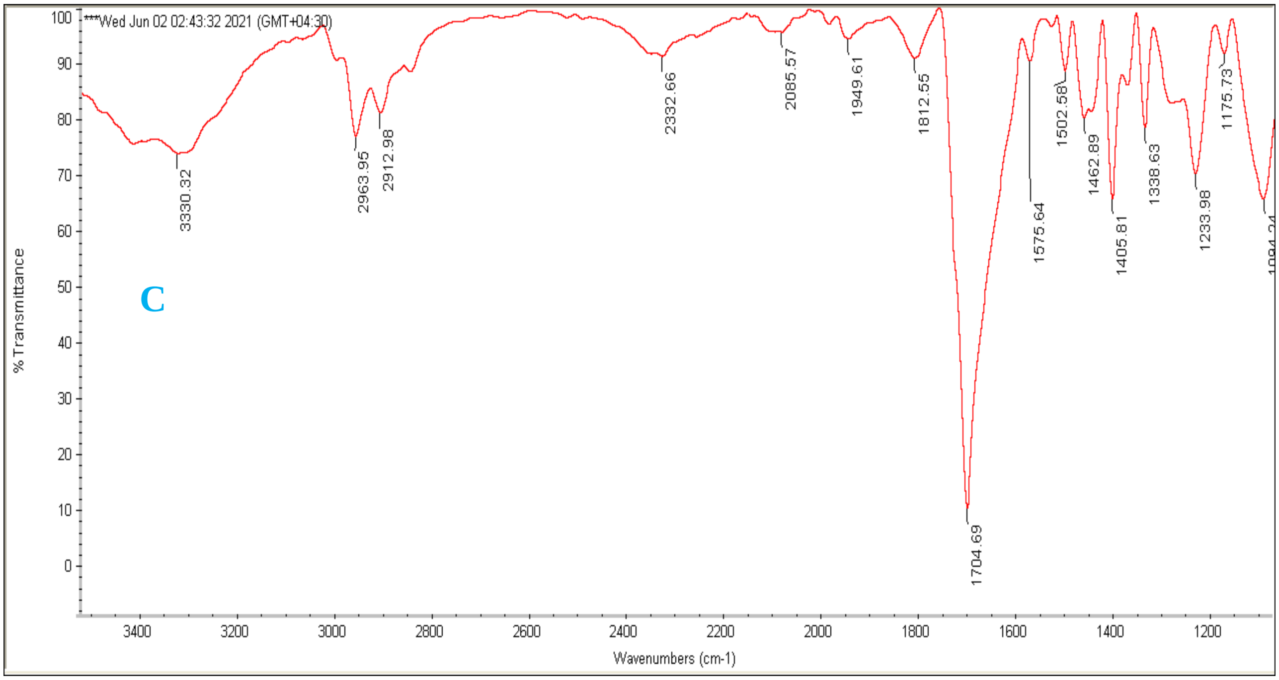

Figure S12. FT-IR spectra of (**A**) Bare ITO-PET, (**B**) ITO-PET-OH-AuNPs, and (**C**) ITO-PET-OH-AuNPs-aptamer-RIV, respectively.

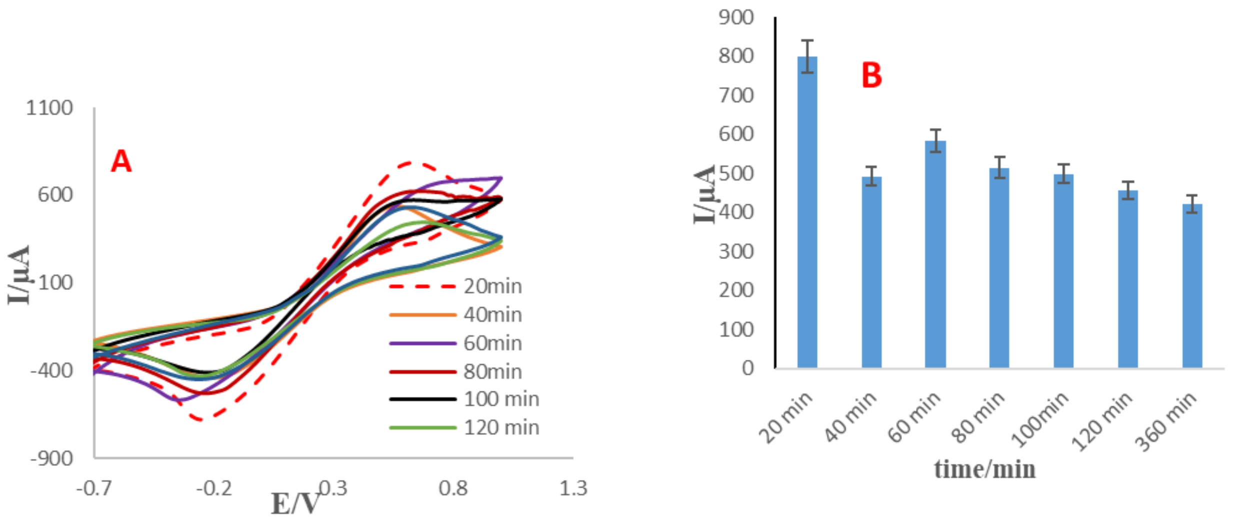

Figure S13. Optimization of various incubation time (20, 40, 60, 80, 100,120 and 360 min) for aptamer immobilization (A) CVs of ITO PET-OH-Au NPs-aptamer in ferrocyanide/ferricyanide/KCl (0.01 M) at the potential rang of −1 to +1 V, sweep rate: 100 mv/s (B) Histogram of peak current *versus* time of incubation. (n=3)

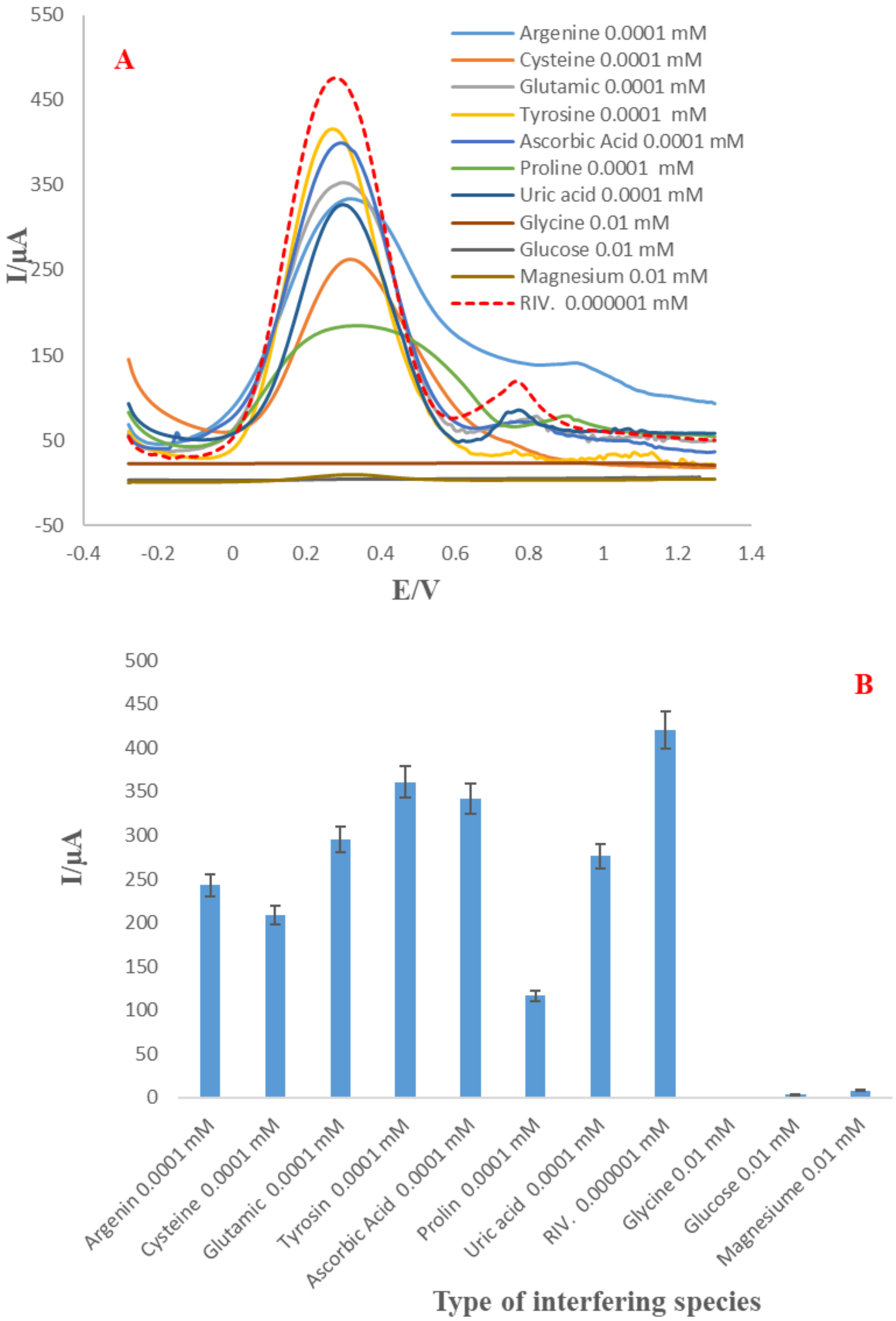

Figure S14. A) SWV of aptasensor in the presence of different interfering agents. **B)** Variation of peak currents *versus* type of interfering species. Supporting electrolyte is 0.01 M $[Fe(CN)_6]^{3-/4-}$, SWV parameters: potential range of -1 V to +1 V with a step potential of 0.01 mV, amplitude 0.25 mV and frequency 1 Hz.

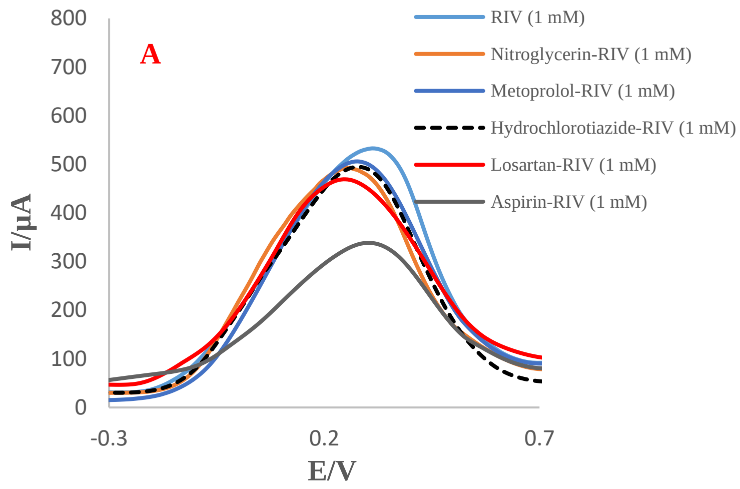

B

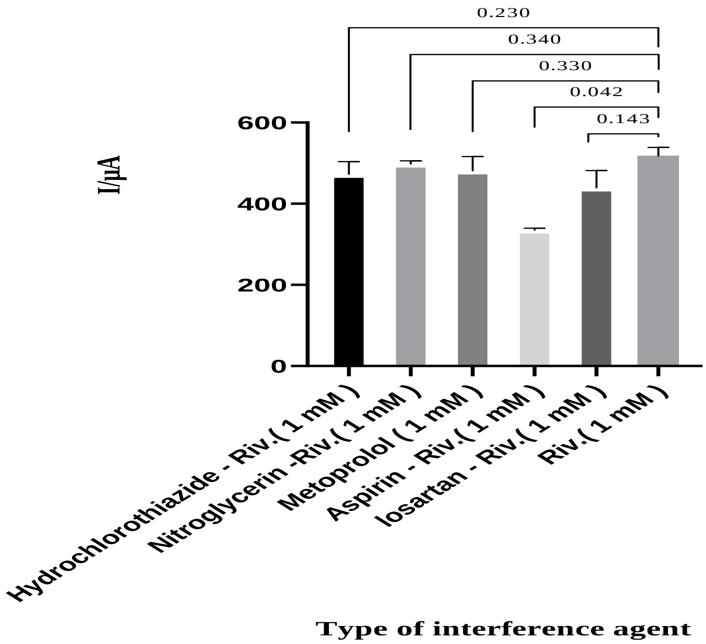

Figure S15. A) SWV of aptasensor in the presence of different species (hydrochlorothiazide, metoprolol, nitroglycerin, atorvastatin, aspirin, and losartan). B) Dependency of peak currents of aptasensor *versus* the type of interfering agents. The concentrations of analogs were 1 mM. Supporting electrolyte is 0.01 M $[Fe(CN)_6]^{3-/4-}$, SWV parameters: potential range of -1 V to +1 V with a step potential of 0.01 mV, amplitude 0.25 mV, and frequency 1 Hz. Statistical significance was determined by Paired t-test. The aspirin shows a significantly lower response than other drugs ($p < 0.05$) indicating significant differences relative to displayed bars as determined.

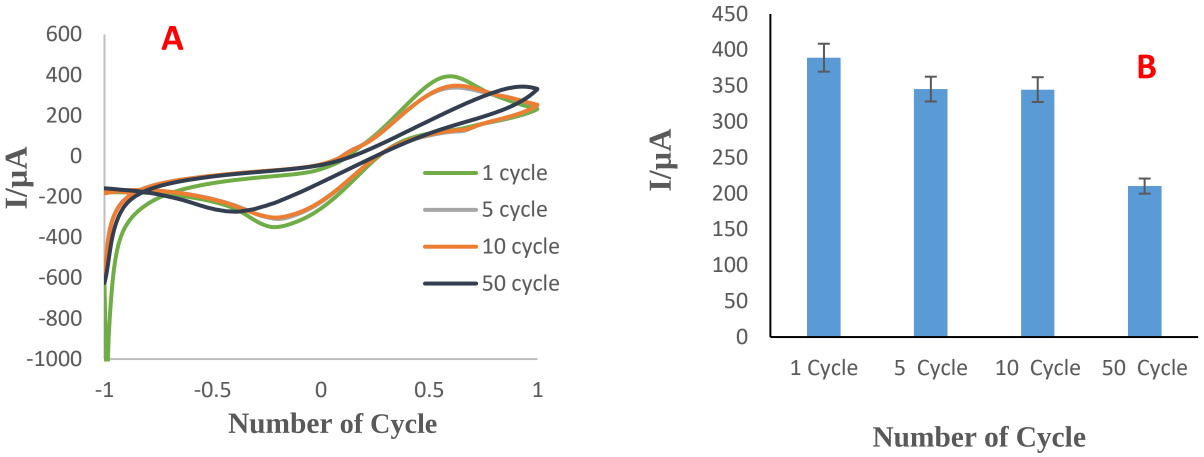

Figure S16. A) CVs of ITO-PET-OH-AuNPs in different cycle number (1-50 Cycle). **B)** Histogram of peak current *versus* number of cycles. Sweep rate is 100 mV/s. supporting electrode is $[Fe(CN)_6]^{3-/4-}$/KCl (0.01 M)]. (n=3).

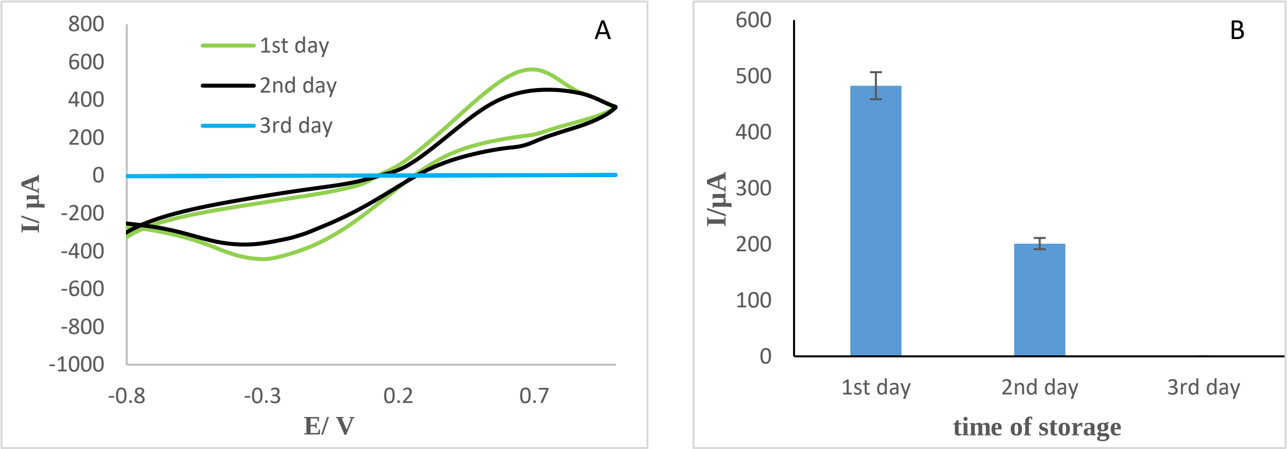

Figure S17. A, CVs of ITO-PET-OH-AuNPs/Aptamer in the various storage time (24, 48, and 72 h) in supporting electrolyte $[Fe(CN)_6]^{3-/4-}$/KCl (0.01 M)] sweep rate is 100 mv/s. (1–3 days). (n=3). B) Histogram of peak current *versus* time of Storage.

Table S1. Validation data of proposed method for quantification of RIV in exhaled breath condensate (EBC) and human plasma samples (n=3).

Parameters	EBC	Plasma
Linear range (nM)	10-600	10-600
Slope	0.1609	0.196
Intercept	90.163	38.356
Correlation coefficient	0.9952	0.9941
Number of data points	7	8
LOD (nM)	6.03	14.08
LOQ (nM)	19.9	46.47

Table S2. Recovery of RIV from exhaled breath condensate (EBC) and plasma samples (n=3). Data are presented as mean ± relative standard deviation (RSD)

plasma and EBC samples	Added RIV (nM)	EBC			Plasma		
		Found (nM)	Recovery (%)	RSD (%) (n=3)	Found (nM)	Recovery (%)	RSD (%) (n=3)
1	10	7.6 (± 3.7)	88.7	6.1	22 (± 2.1)	107.7	4.9
2	50	57.9 (± 2.2)	101.8	2.3	46.5 (± 2.5)	108.1	5.0
3	100	118.5 (± 0.5)	104.3	0.4	95.6 (± 1.3)	99.5	2.3
4	300	289 (± 1.9)	98.9	1.4	280.3 (± 1.3)	96.2	2.0
5	500	494.7 (± 2.2)	99.5	1.3	498.7 (± 2.2)	99.7	1.6

Table S3. Comparison of the recovery of RIV from exhaled breath condensate (EBC) and plasma samples (n=3), and the relative standard deviations (RSD %) analyzed by the proposed aptasensor and LC-MS/MS method

Sample (nM)	EBC Recoveries (%)		Plasma Recoveries (%)	
	Aptasensor	LC-MS/MS	Aptasensor	LC-MS/MS
10	88.7 (± 6.1)	107.0 (± 0.21)	107.7 (± 4.9)	103.3 (± 2.1)
50	101.8 (± 2.3)	105.7 (± 0.2)	108.1 (± 5.0)	105.4 (± 0.8)
100	104.3 (± 0.4)	100.2 (± 0.25)	99.5 (± 2.3)	98.9 (± 0.4)
300	98.9 (± 1.4)	96.7 (± 0.06)	96.2 (± 2.0)	99.9 (± 0.2)
500	99.5 (± 1.3)	101.0 (± 0.08)	99.7 (± 1.6)	99.7 (± 0.1)

Table S4. The repeatability of aptasensor

Concentration(nM)	Ip1	Ip2	SD	AVE_{STDV}
10	278	276	1.41	
50	300	295	3.53	
100	321	317	2.82	
300	411	414	2.12	
500	474	480	4.24	2.8
